# Enhanced Photocatalytic Performance of Halogenated Phenylacetylene-Decorated Cu_2_O Surfaces via Electronic Structure Modulation: A DFT and Experimental Study

**DOI:** 10.1021/acsnanoscienceau.5c00030

**Published:** 2025-06-05

**Authors:** Jui-Cheng Kao, Wei-Yang Yu, Kuo-Chang Chien, Po-Jung Chou, Michael H. Huang, Yu-Chieh Lo, Jyh-Pin Chou

**Affiliations:** † Department of Materials Science and Engineering, 34914 National Yang Ming Chiao Tung University, Hsinchu 30010, Taiwan; ‡ Department of Chemistry, Frontier Research Center on Fundamental and Applied Sciences of Matters, 34881 National Tsing Hua University, Hsinchu 30013, Taiwan; § Graduate School of Advanced Technology, 33561 National Taiwan University, Taipei 106319, Taiwan

**Keywords:** Cu_2_O, photocatalysis, density functional theory, surface modification, band structure modulation

## Abstract

This study investigates the photocatalytic performance of Cu_2_O surfaces modified with halogen-substituted phenylacetylenes (4-XA), including 1-ethynyl-4-fluorobenzene (4-FA), 1-chloro-4-ethynylbenzene (4-CA), and 1-bromo-4-ethynylbenzene (4-BA), using an integrated theoretical and experimental approach. Through density functional theory (DFT) calculations and ultraviolet photoelectron spectroscopy (UPS) measurements, we analyze how these molecular decorators affect charge transfer dynamics and the electronic structure of the Cu_2_O {100}, {110}, and {111} facets. Two distinct photocatalytic mechanisms are proposed: one where electrons reach the vacuum level through the molecular decorator and another where electrons escape directly through the Cu_2_O surface via molecular-induced hybridized states. Our results show that 4-BA-modified {100} surfaces exhibit the strongest enhancement, which is attributed to the presence of in-gap molecular states, increased charge separation, and a significantly reduced work function. Experimental degradation of methyl orange validates the trend 4-BA > 4-CA > 4-FA, consistent with theoretical predictions. These findings highlight the crucial role of band structure engineering and provide guidelines for the rational design of high-performance molecularly decorated photocatalysts.

## Graphical abstract

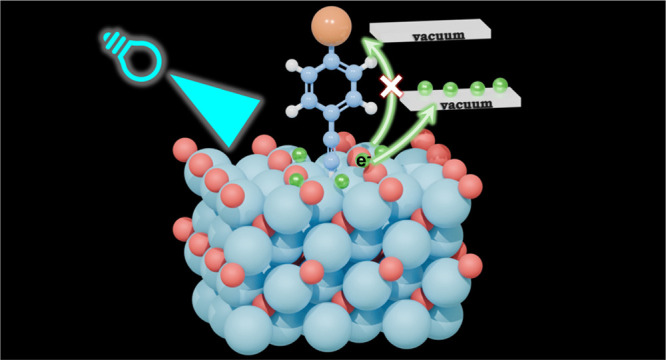

## 1 Introduction

Photocatalysts have received considerable attention over the past few decades due to their extensive applications in areas such as air and water purification, green chemistry, active surface engineering, and energy conversion.^1–8^ Enhancing photocatalytic activity for processes such as dye degradation,^9,10^ water splitting,^11–13^ CO_2_ reduction,^14,15^ and organic transformation reactions^16,17^ remains a critical goal for advancing sustainable technologies. A comprehensive understanding of the reaction mechanism is pivotal in the design of highly efficient photocatalytic materials.

For many reported photocatalysts employed in water splitting,^18–20^ reactants must adsorb onto the surface to proceed with subsequent reaction steps. This prerequisite highlights the role of diffusion from the bulk aqueous environment and mass transport as limiting factors in these processes. Conversely, these constraints can be effectively addressed by leveraging free-radical-based mechanisms, which are particularly advantageous for organic reactions. In these systems, photoexcited electrons transfer to dissolved oxygen, generating superoxide anion radicals that subsequently react with organic molecules.^21,22^ Meanwhile, numerous semiconductor materials, including Cu_2_O, CeO_2_, Ag_2_O, Ag_3_PO_4_, and SrTiO_3_, display strong facet dependence in photocatalytic activity.^23–27^ For example, Cu_2_O rhombic dodecahedra (RD) manifest high photocatalytic activity, while cubes are simply inert due to the lack of radical formation upon light illumination.^23^ This phenomenon can be explained by density functional theory (DFT) calculations, which reveal that the facet dependence of Cu_2_O originates from its electronic structure.^28^

To enhance photocatalytic activity, various strategies have been explored, including surface decoration with metal particles and graphene sheets, graphitic carbon nitride (g-C_3_N_4_), and the formation of semiconductor heterostructures.^23,29–34^ DFT calculations demonstrate that functionalization of Cu_2_O crystals with specific adsorbates can significantly modify their band structures, enhancing their suitability for photocatalytic applications.^28^ For example, molecular functionalization of Cu_2_O cubes with 4-ethynylaniline (4-EA) has been shown to transform otherwise inert cubes into highly active photocatalysts.^35^ Cu_2_O octahedra and RD also exhibit modest activity improvements upon 4-EA decoration. This enhancement is attributed to the creation of new electronic states within the band gap, as confirmed by band-decomposed charge density analyses. Among the molecular decorators studied, 4-trifluoromethylphenylacetylene (4-TFMA) has demonstrated unique ability to enhance photocatalytic performance. Decoration of Cu_2_O surfaces with 4-TFMA significantly improves photocatalytic activity on {100} and {110} facets due to the introduction of in-gap states and enhanced charge separation. However, the {111} surface shows diminished performance upon 4-TFMA decoration, primarily due to the absence of in-gap states and electron localization effects between the 4-TFMA molecule and the Cu_2_O surface.^36^ These findings highlight the facet-dependent nature of 4-TFMA-induced photocatalytic activity and its potential as a cost-effective and efficient molecular decorator.

The use of alternative molecular decorators has provided deeper insights into photocatalytic mechanisms. Functionalization with 2-ethynyl-6-methoxynaphthalene (2E-6MN) leads to distinct photocatalytic behaviors, with notable changes in band structure for {100} and {110} surfaces, while no new bands appear within the band gap for the {111} surface.^37^ Similarly, 4-nitrophenylacetylene (4-NA) enhances photocatalytic performance by facilitating electron injection and achieving a small energy difference between the 4-NA molecule and the vacuum level, as corroborated by DFT calculations.^38^ These findings underscore the potential of molecular decoration in tailoring photocatalytic activity for specific applications, such as aryl sulfide oxidation,^39^ oxidative amine coupling reactions,^40^ and arylboronic acid hydroxylation.^41^ Furthermore, Bader charge difference and charge density analyses reveal that 4-cyanophenylacetylene (4-CNA) acts as an efficient electron transfer pathway, facilitating electron migration away from the Cu_2_O surface.

Ligand-associated semiconductor nanoparticles are outstanding choices for organic coupling reactions^21,22^ under the balance between the cost and economic efficiency compared to other materials such as metal organic frameworks and covalent organic frameworks.^42–44^ These molecular decorators can be broadly classified into two groups based on their electronic behavior: electron-donating (e.g., 4-EA, 2E-6MN) and electron-withdrawing (e.g., 4-NA, 4-CNA, 4-TFMA). Although both types enhance photocatalytic activity, experimental findings reveal that electron-withdrawing groups generally result in stronger activity improvements on Cu_2_O {100} surfaces.^38,41^

In this study, we investigate halogen-substituted phenylacetylenes as molecular decorators, focusing on 1-ethynyl-4-fluorobenzene (4-FA), 1-chloro-4-ethynylbenzene (4-CA), and 1-bromo-4-ethynylbenzene (4-BA). These halogen substituents were deliberately chosen to systematically probe the effects of substituent electronegativity and resonance interaction on Cu_2_O band structure modulation. They offer a simplified and tunable framework for studying how molecular properties influence interfacial charge transfer. Fluorine, chlorine, and bromine offer a progressive decrease in electronegativity, which can influence the degree of electron-withdrawing interaction formation at the molecule–surface interface. Using DFT calculations, we evaluate the charge transfer behavior on Cu_2_O {100}, {110}, and {111} surfaces through charge density difference, Bader charge analysis, planar average potential distribution, and energy band diagrams. Our results reveal the formation of new molecular-induced bands within the band gap after surface decoration, significantly enhancing photocatalytic performance. Furthermore, our calculations predict that the photocatalytic efficiency follows the trend 4-BA > 4-CA > 4-FA, consistent with our experimental results. Notably, we propose a novel mechanism for decorated Cu_2_O surface systems, wherein electrons preferentially transition to vacuum-level states through the Cu_2_O surface rather than via molecular decorators. This mechanism diverges from previous findings.^35–38,41^ Through a combination of theory and experiment, we identify two key factors influencing photocatalytic enhancement: (i) the emergence of molecular-induced hybrid states near the band edge and (ii) the modified surface electronic potential that facilitates electron escape. These insights offer a new mechanistic understanding of facet-specific molecular decoration and provide a rational basis for designing next-generation photocatalytic systems.

## 2 Results and Discussion

### 2.1 Electronic Comparison of Decorators

To determine the nature of the three molecules (4-BA, 4-CA, 4-FA) prior to decoration, we analyzed their molecular electrostatic potential (MEP) and electron localization function (ELF). Figure 1 presents the MEP and ELF distributions for these molecules. In the MEP analysis, the isosurface distribution represents the charge density, while the color indicates the electrostatic potential. The bromo substituent exhibits a green-to-blue color gradient (see Figure 1a), indicating a lower electrostatic potential compared to the fluoro substituent, which predominantly appears red (see Figure 1c). Notably, the electrostatic potential follows the trend: F– > Cl– > Br–. This observation suggests that the electron-withdrawing effect is strongest for the bromo group and weakest for the fluoro group. Additionally, the isosurface analysis reveals that the bromo substituent has the highest charge density among the three molecules. Subsequently, the ELF analysis quantifies the likelihood of electron localization around specific atoms. Results show that the bromo substituent exhibits the highest probability of electron localization, followed by the chloro and fluoro substituents, which display weaker electron-withdrawing behavior. Integrating the MEP and ELF findings, we conclude that the electron-withdrawing property of the substituents follows the sequence: 4-BA > 4-CA > 4-FA. Interestingly, this trend is opposite to their electronegativity values, highlighting the unique electronic environment created by the bromo group.

**Figure 1:**
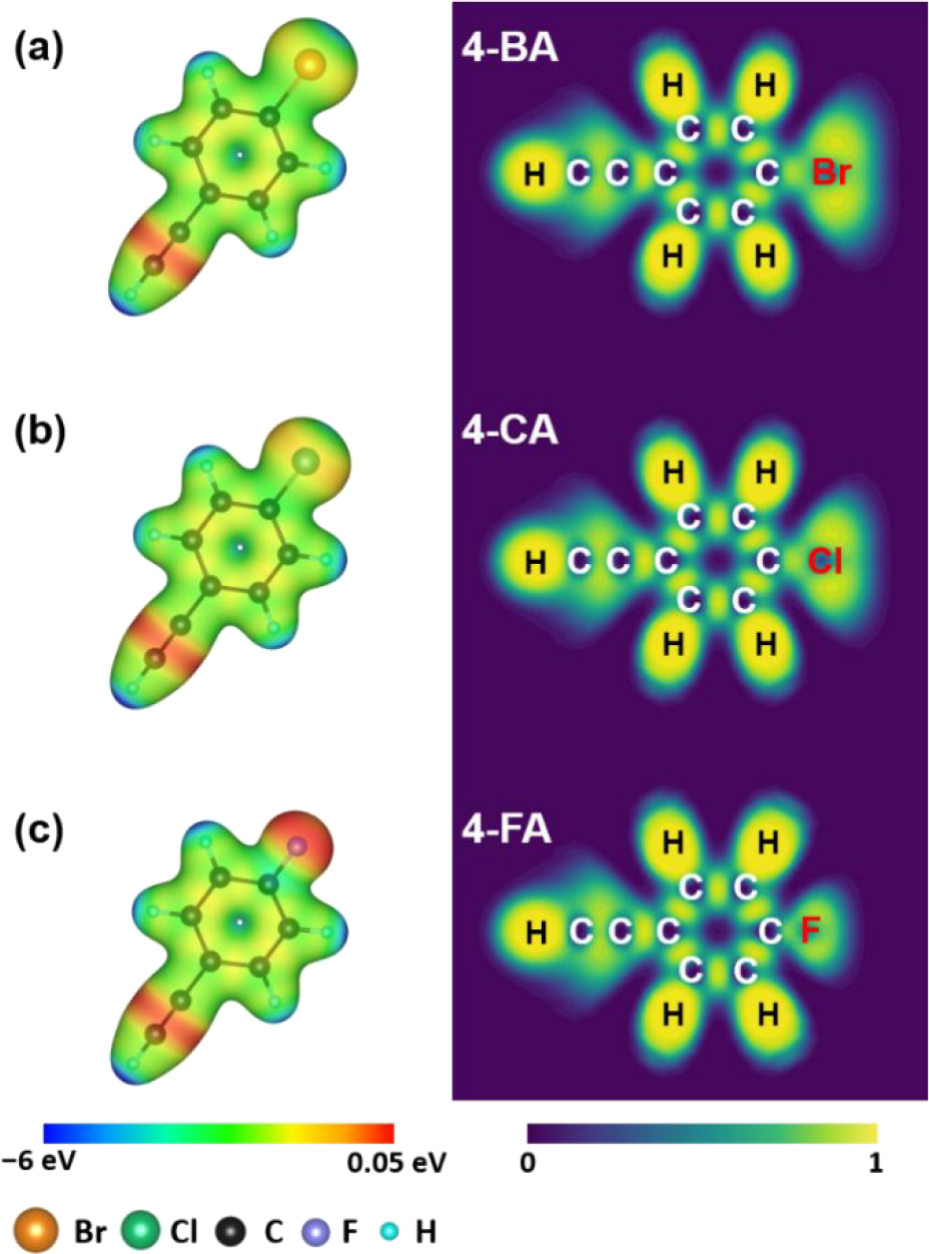
Molecular electrostatic potential (left panels) and 2D presentation of the electron localization function (right panels) of (a) 4-BA, (b) 4-CA, and (c) 4-FA. The isosurface value of the charge density is set to 0.025 e/Bohr^3^.

Halogen substituents withdraw electrons through the inductive effect due to their high electronegativity, while their lone pairs may also engage in resonance with the benzene ring, partially offsetting this effect. Among them, fluorine shows the strongest resonance interaction owing to its well-matched 2*p* orbital, weakening its net electron-withdrawing ability. In contrast, bromine exhibits the weakest resonance effect, and despite its relatively low electronegativity, it achieves the strongest overall electron-withdrawing capacity. Consequently, the electron-withdrawing ability follows the trend: 4-BA > 4-CA > 4-FA. This trend enhances photocatalytic performance, as the stronger withdrawal effect of 4-BA facilitates charge separation and suppresses electron–hole recombination under illumination. By integrating the MEP and ELF results, we find the strong potential of these molecular properties to influence photocatalytic performance. Given its superior electron-withdrawing behavior, 4-BA is expected to significantly enhance the photocatalytic activity of Cu_2_O crystals. These predictions are further explored in subsequent sections.

### 2.2 Decorated Cu_2_O {100} Surface

The decorated structures for the Cu_2_O {100} surface are demonstrated in Figure S1. Due to the metal cation−π interactions and the disappearance of the acetylenic hydrogen featured peak of the functionalized Cu_2_O crystal observed in the Fourier-transform infrared (FT-IR) spectra from previous studies,^36–38,41^ the H atom of alkynyl termination needs to be removed for the decorators. For the {100} surface, there are four bridge sites based on the different Cu–Cu and O–O distances, which are denoted as Cu–Cu long-range, Cu–Cu short-range, O–O long-range, and O–O short-range (see Figure S1d–f). The most stable binding site for three decorating molecules is the Cu–Cu long-range bridge site. The corresponding binding energies for 4-FA-, 4-CA-, and 4-BA-decorated Cu_2_O {100} surfaces are −3.95 −3.98, and −3.95 eV, respectively. These considerable negative values mean that the binding between the Cu_2_O {100} surface and three decorating molecules is relatively stable. The detailed analysis of the binding configurations and the corresponding binding energies can be seen in the Supporting Information.

Following the determination of stable binding configurations, the behavior of charge transfer between the semiconductor surface and the conjugated molecule would be investigated. As seen in Figure 2a, the planar average charge density difference (CDD), along the surface normal for the 4-BA-decorated Cu_2_O {100} surface, and the side view in the 3D representations are provided. The red dashed line in the plot is used to separate the Cu_2_O surface from the 4-BA molecule, which is defined as the middle of the alkynyl carbon atom and the binding Cu atom. The integral values for the upper side and lower side separated by the red dashed line are 1.341e and −1.592e, respectively. The positive value means charge accumulation at the 4-BA molecule, whereas the negative value signifies charge depletion for the Cu_2_O surface. Therefore, the CDD result illustrates that the electron tends to transfer from Cu_2_O to the 4-BA molecule. The CDD calculations for 4-FA- and 4-CA-decorated Cu_2_O {100} surfaces are presented in Figure S3, which also suggest a similar result that electrons are inclined to transfer from the Cu_2_O surface to decorating molecules. To further compare the level of charge separation in the decorated Cu_2_O {100} surface, the integral values of CDD are also provided. The values for 4-BA-, 4-CA-, and 4-FA-decorated surfaces are 1.176e, 1.160e, and 1.152e, respectively. These values suggest that 4-BA promotes the most effective charge separation, aligning with its anticipated superior photocatalytic activity. To quantify the investigation of charge transfer behavior, Bader charge difference (BCD) calculations are also performed. The BCD values for decorating molecules are 0.036e, 0.050e, and 0.049e for 4-BA, 4-CA, and 4-FA cases, respectively, indicating that decorating molecules tend to obtain electrons. Besides, the BCD values for the Cu atoms bonded to decorating molecules are −0.160e for three cases, signifying that electrons are inclined to transfer out of Cu_2_O surfaces.

**Figure 2:**
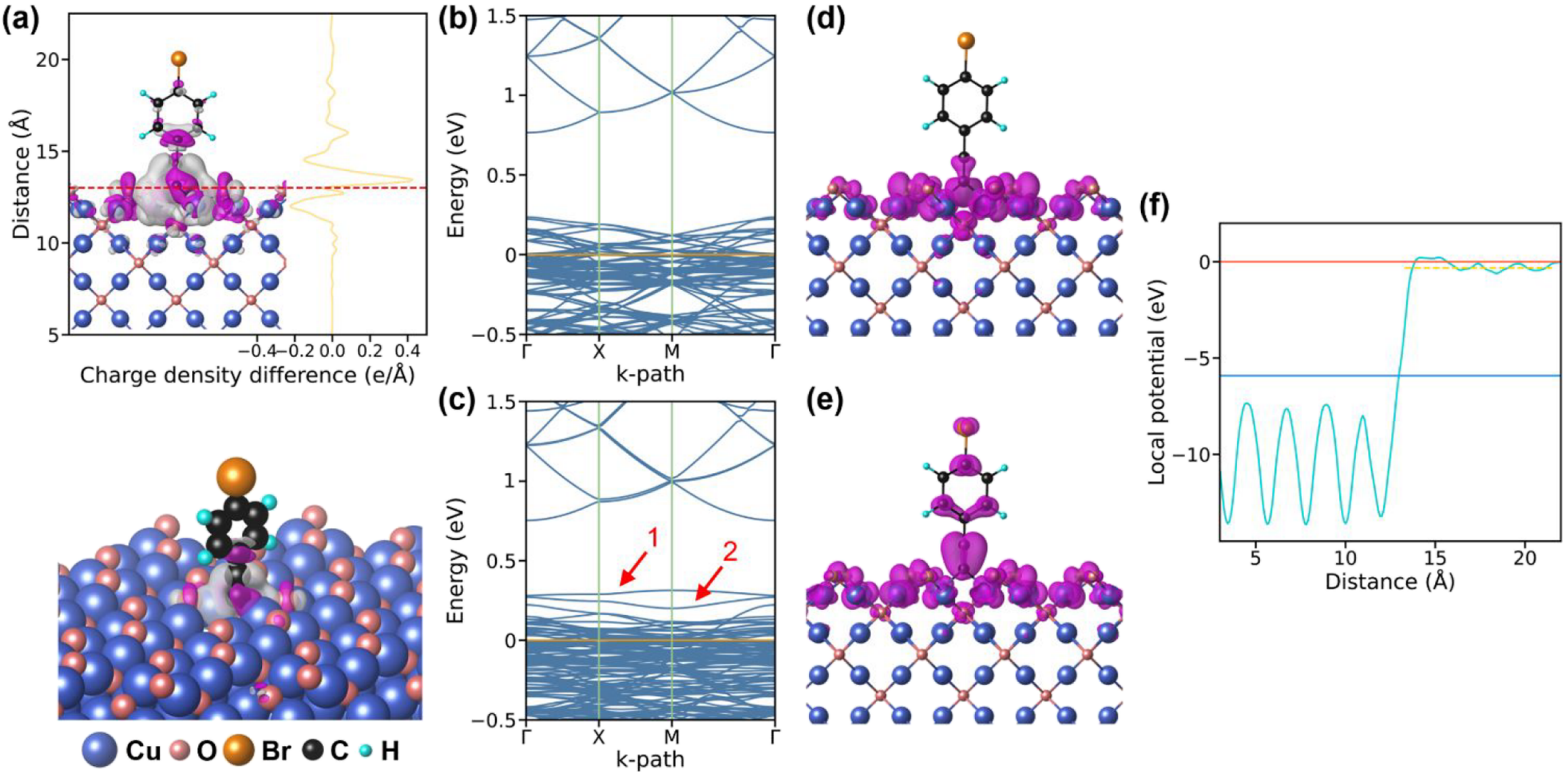
(a) Planar average charge density difference (CDD) along the surface normal for the Cu_2_O {100} surface decorated with a 4-BA molecule and the side view in the 3D representations (upper panel). The oblique view of the CDD for the Cu_2_O {100} surface decorated with 4-BA molecules (lower panel). The isosurface value is set to 0.001 e/Bohr^3^. The purple and gray regions represent charge accumulation and depletion, respectively. Band structure of (b) the pristine Cu_2_O {100} surface and (c) decorated with a 4-BA molecule. The band-decomposed charge density of the (d) higher (denoted as 1) and (e) lower (denoted as 2) 4-BA-induced band within the band gap for the {100} surface. The orange lines are Fermi level, which are all aligned to zero. (f) Planar average electrostatic potential along the surface normal for the Cu_2_O {100} surface decorated with a 4-BA molecule. The red and blue solid lines are denoted as vacuum level and Fermi level, respectively. The yellow dashed line represents the average potential of the decorating molecule.

Next, molecular functionalization should tune the surface band structure of Cu_2_O to display different photocatalytic activities.^35–38,41^ Figure 2b,c presents the band structure of the Cu_2_O {100} surface before and after 4-BA molecule decoration. Apparently, there are two new molecular-induced bands located between the band gap after 4-BA decoration, denoted as band-1 and band-2, respectively. These molecularly induced bands do not exhibit the characteristics of deep trap states, which typically localize charge and hinder transport. Instead, band-decomposed charge density analysis reveals that band-1 and band-2 possess hybridized characteristics, with charge density delocalized over the Cu_2_O surface and partially extended into the molecular region (as shown in Figure 2d,e). This spatial distribution suggests that these states act as intermediate “*electron bridges*,” promoting directional charge transfer toward the vacuum level. Such hybridization lowers the energy barrier for electron escape and enhances charge separation, thereby improving the photocatalytic efficiency. More specifically, the charge density of band-1 is concentrated near the junction between the Cu_2_O surface and the 4-BA molecule, while band-2 exhibits broader delocalization across both the molecule and part of the surface. The projected band structures of the Cu_2_O {100} surface decorated with 4-BA are shown in Figure S4. Within the energy range of −0.5 to 1.5 eV, there is no notable contribution from H atoms. Band-1 is mainly composed of surface Cu and -O atoms, while the C atom contribution is minor. Similarly, band-2 also originates primarily from Cu and O atoms, with a small contribution from the C atom and a slight contribution from the Br atom. Figure S5 demonstrates the band structure and band-decomposed charge density calculation results for 4-FA- and 4-CA-decorated {100} surfaces, which are similar to those in the 4-BA case. Hence, these two new molecular-induced bands can be treated as another energy-efficient pathway for electrons to escape from Cu_2_O surfaces, thus optimizing the photocatalytic activity.

Another way to evaluate photocatalytic activity is a comparison of the work function, as a smaller work function results in preferable photocatalytic activity.^45^ Work function is a key parameter influencing the surface electron transfer and photocatalytic efficiency. While it is commonly associated with hole energetics in the valence band, its effect on electron mobility is equally critical. A reduction in the work function lowers the energy barrier for electron escape, promoting enhanced charge separation and reducing recombination rates. In our study, the observed decrease in work function upon molecular decoration correlates with improved photocatalytic activity, supporting the hypothesis that tuning the work function can be an effective strategy for optimizing photocatalytic performance. Figure 2f portrays the planar average electrostatic potential along the surface normal for the 4-BA-decorated Cu_2_O {100} surface with a work function of 5.91 eV (i.e., energy difference between the vacuum level and Fermi level). Compared to the value of the pristine Cu_2_O {100} surface, which is 6.88 eV, the 4-BA decorated Cu_2_O {100} surface displays a considerable decrease in work function to largely improve the photocatalytic performance. Although work function and ionization energy are ground-state properties, their variation reflects the absolute shift of energy levels relative to the vacuum. Since photocatalysis involves photoexcited electrons in the conduction band, a reduced work function corresponds to a CBM closer to the vacuum level, lowering the escape barrier for electrons. While the CBM position relative to the Fermi level remains nearly unchanged (Figure 2b,c), the absolute CBM energy decreases, in line with the work function, explaining the enhanced photocatalytic activity upon molecular decoration. The work function values for 4-FA- and 4-CA-decorated Cu_2_O {100} surfaces are provided in Figure S6, which are 5.99 and 5.94 eV, respectively. Thus, the sequence of work function follows the pattern: 4-BA < 4-CA < 4-FA, while the efficiency for photocatalytic activity is opposite: 4-BA > 4-CA > 4-FA. Besides, we also calculated the average potential of the 4-BA molecule (orange dashed line in Figure 2f), which is very close to the vacuum level. The counted range is from 13.35 to 20.13 Å, which is the same as the length of the 4-BA molecule. The potential difference between this molecule level and the vacuum level is 0.31 eV, elucidating that electrons are easier to transfer out to the vacuum for further radical formation after reaching the 4-BA molecule. However, it seems that electrons face a substantial energy barrier to reach the 4-BA molecule, which can be seen in the plot where the potential range from approximately 13.35 to 15.13 Å exceeds the vacuum level. This phenomenon can affect the outcome of the photocatalytic activity. A similar situation can be found for 4-FA- and 4-CA-decorated Cu_2_O {100} surfaces, which are presented in Figure S6.

### 2.3 Photocatalytic Mechanism

Previous studies^35–38,41^ have suggested that the photocatalytic mechanism of the functionalized Cu_2_O surfaces follows the schematic depicted in Figure 3a. DFT calculations reveal that new molecular decoration with species such as 4-EA, 2E-6MN, 4-NA, 4-CNA, and 4-TFMA introduces new molecular-induced electronic states within the band gap. These states facilitate electron transfer from the Cu_2_O surface to the decorating molecules, enabling the decorating molecule to serve as an intermediary “springboard” for photoinduced electrons to reach the vacuum level and participate in subsequent photodegradation reactions under light illumination.

**Figure 3:**
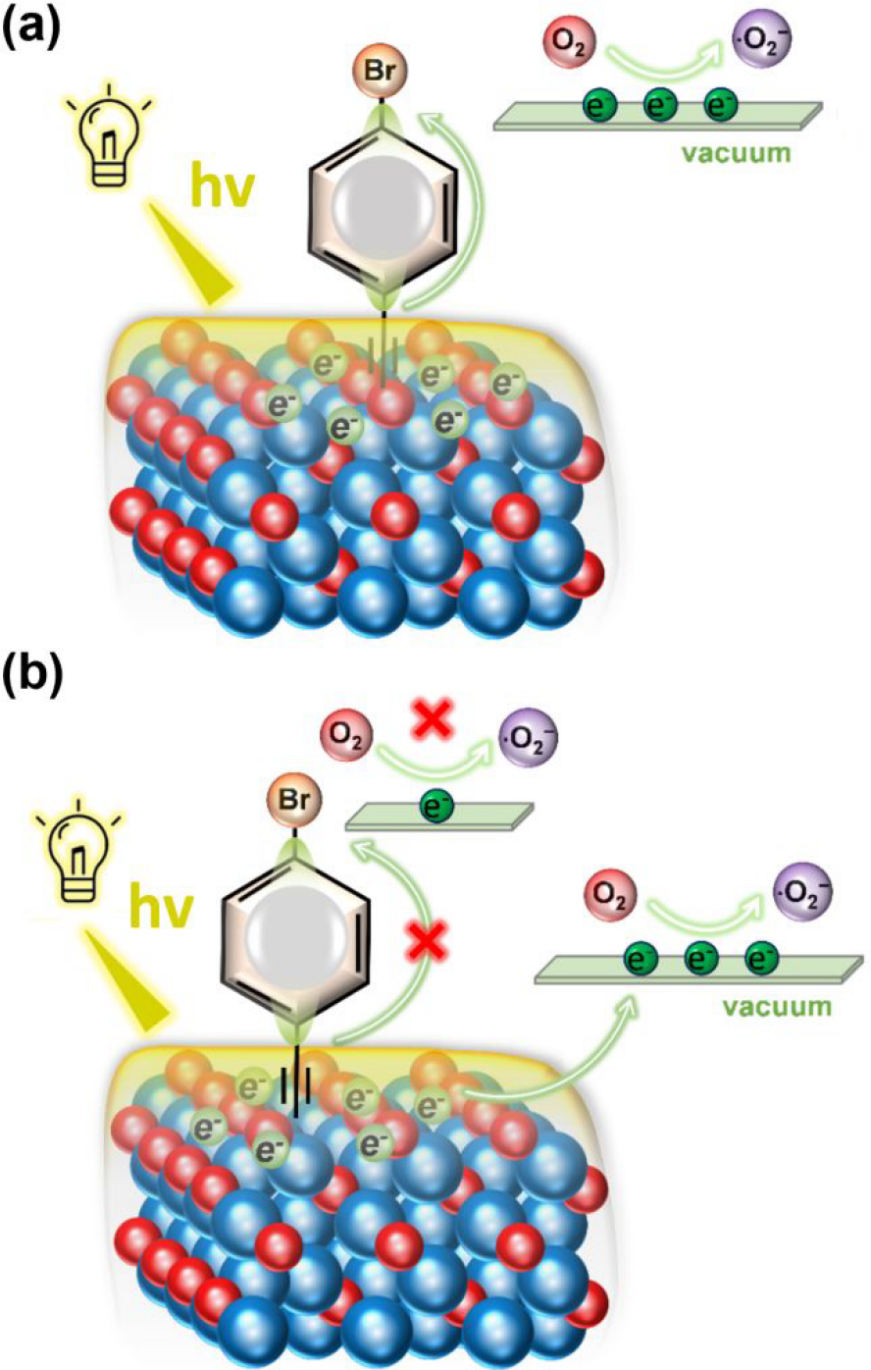
Schematic diagram of two possible photocatalytic mechanisms.

However, our study proposes a distinct photocatalytic mechanism, as illustrated in Figure 3b. Several compelling pieces of evidence support this revised mechanism. First, the analysis of the potential beyond the vacuum level at the interface of the 4-BA-decorated Cu_2_O {100} surface (Figure 2f) and the exceptionally small BCD value for the 4-BA molecule (0.036e) indicates that photoinduced electrons face significant difficulty transferring to the 4-BA molecule. Additionally, band-decomposed charge density analyses provide further insights. The charge density corresponding to band-1, which is located higher in energy than band-2 in the band structure, is distributed primarily around the Cu_2_O surface (Figure 2c,d). This finding implies that electrons accumulating near the Cu_2_O {100} surface can reach the vacuum level with a lower energy barrier, bypassing the need to transfer them to the 4-BA molecule.

Thus, the proposed mechanism suggests that electrons do not preferentially reach the vacuum level through the decorating molecule. Instead, for the 4-BA-decorated Cu_2_O {100} surface, the 4-BA molecule facilitates a novel pathway for electrons to escape via the Cu_2_O surface itself. This alternative mechanism highlights the critical role of molecular decoration in modifying the electronic landscape of semiconductors and enabling more efficient photocatalytic processes.

To elucidate the energy level shifts of the frontier orbital before and after molecular decoration, Figure 4 shows a comparative band diagram for Cu_2_O {100} surfaces, both undecorated and decorated with 4-FA, 4-CA, and 4-BA. The red and blue bars represent the conduction band and valence band, respectively. The values of the conduction band minimum (CBM) and valence band maximum (VBM) suggest two important factors that directly affect the photocatalytic activity, which are ionization energy and electron affinity. First, ionization energy, defined as the energy difference between VBM and vacuum level, reflects the difficulty for electrons to escape from the valence band. Analogous to work function, a higher ionization energy means fewer electrons can reach the vacuum level, thereby diminishing photocatalytic activity. The ionization energy follows the order: 4-FA (5.77 eV) > 4-CA (5.72 eV) > 4-BA (5.69 eV); thus the 4-BA molecule makes a great figure in photocatalytic performance. In our proposed photocatalytic mechanism, molecular decoration, particularly with 4-BA, facilitates a novel pathway for electrons to escape via the Cu_2_O surface. A lower ionization energy, as observed with 4-BA decoration (5.69 eV compared to 6.66 eV for the undecorated surface), indicates a reduced energy barrier for electrons to reach the vacuum level, enhancing surface electron transfer and photocatalytic activity. This is consistent with our findings that the charge density is primarily distributed around the Cu_2_O surface, enabling electrons to reach the vacuum level without transferring to the decorating molecule.

**Figure 4:**
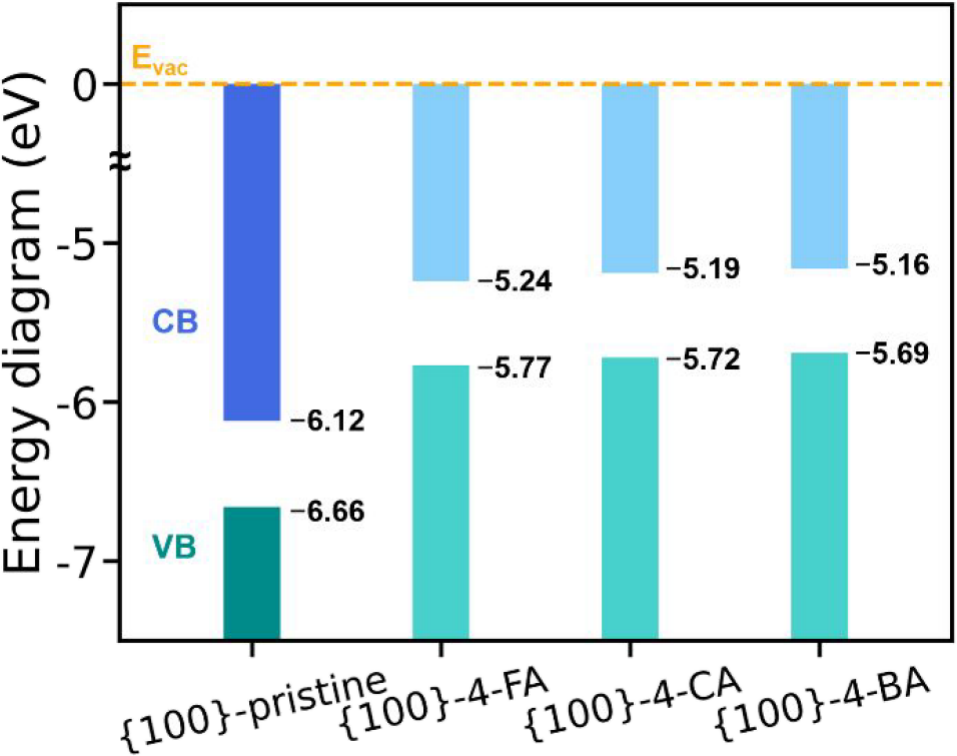
Band diagram for the Cu_2_O {100} surface before and after 4-FA, 4-CA, and 4-BA decoration. *E*_vac_ stands for the energy of the vacuum level and is aligned to zero (orange dashed line). The upper and lower values represent the CBM and VBM, respectively.

Second, electron affinity, defined as the energy difference between the CBM and vacuum level, reflects the energy released when an electron transitions from the vacuum level back to the Cu_2_O surface. A higher electron affinity increases the likelihood of electrons returning to the surface, resulting in a shorter lifetime in the vacuum level and reduced photocatalytic activity. The order of the electron affinity is 4-FA (5.24 eV) > 4-CA (5.19 eV) > 4-BA (5.16 eV), further highlighting the advantage of 4-BA in photocatalytic performance due to its lower electron affinity. Furthermore, a comparison of the Cu_2_O surface before and after molecular decoration shows significant reductions in both ionization energy (from 6.66 eV to lower values) and electron affinity (from 6.12 eV to lower values). These decreases contribute to improved photocatalytic performance by facilitating electron ejection and reducing recombination likelihood. The observed trends underscore the effectiveness of molecular functionalization, particularly with 4-BA, in optimizing the electronic properties of Cu_2_O {100} surfaces for enhanced photocatalytic applications.

### 2.4 Decorated Cu_2_O {110} and {111} Surfaces

Figure S2 demonstrates the binding structures for Cu_2_O {110} and {111} surfaces decorated with a 4-FA molecule. The most stable binding sites for both cases are top sites, and the corresponding binding energies for 4-FA-decorated Cu_2_O {110} and {111} surfaces are −2.16 and −4.32 eV, respectively. For the 4-BA cases, the binding energies are −2.16 eV and −4.29 eV for the {110} and {111} surfaces, respectively. These large chemisorption energies indicate that both 4-FA and 4-BA bind strongly to the Cu_2_O surfaces. Figure S7 presents the CDD results. The red dashed line is denoted the same as in the Cu_2_O {100} case. We find electron accumulation at the 4-FA molecule and electron depletion at the Cu_2_O surface. The integral values of CDD for 4-FA-decorated {110} and {111} surfaces are 0.872e and 1.275e, respectively. The smaller value for the {110} surface suggests poor charge separation after molecular modification, thus strongly affecting the photocatalytic activity. For the 4-BA-decorated surfaces, the integrated values of CDD are 0.870e and 1.286e for the {110} and {111} surfaces, respectively, which are comparable to those of the 4-FA cases. Bader charge difference shows that the surface Cu atom and the 4-FA molecule both tend to obtain electrons in the case of the decorated {111} surface, reducing the amount of electrons reaching the vacuum level, which impedes its photocatalytic performance. Also, Figure S8 provides the band structure of the 4-FA- and 4-BA-decorated Cu_2_O {110} and {111} surfaces. One 4-FA-induced band exists in the case of the {110} surface after functionalization (see Figure S8b **)**, denoted as band-1, and Figure S8d displays the band-decomposed charge density of band-1, showing the charge localization at the junction between the Cu_2_O surface and 4-FA molecule. Similar band structure and charge density distribution of the molecule-induced band can be seen for 4-BA cases (Figure S8c,e). On the other hand, no new bands appear in the case of the {111} surface after decoration, giving rise to poor photocatalytic enhancement.

To evaluate the ability of electrons to reach the vacuum level and conduct further reactions, work function, electron affinity, and ionization energy are also calculated. Figures S9 and S10 present the planar average potential analysis and energy band diagram for 4-FA- and 4-BA-decorated Cu_2_O {110} and {111} surfaces, respectively. Work functions for 4-FA-decorated Cu_2_O {110} and {111} surfaces are 5.72 and 5.32 eV, respectively. Comparable work function values are shown in 4-BA-decorated surfaces (Cu_2_O {110}-4-BA: 5.73 eV, Cu_2_O {111}-4-BA: 5.35 eV). The work function of the decorated surface remains almost the same as compared to the pristine surface (Cu_2_O {110}: 5.71 eV, Cu_2_O {111}: 5.29 eV) for both the {110} and {111} surfaces. As for electron affinity and ionization energy, it is apparent that the CBM and VBM for decorated Cu_2_O {110} and {111} surfaces deviate from the pristine Cu_2_O {110} and {111} surfaces only by a small margin (see Figure S10). Therefore, we conclude that the ability for electrons leaving the Cu_2_O surface would not differ obviously before and after molecular decoration, expecting slight photocatalytic activity improvement.

### 2.5 Experimental Validation

Photocatalytic experiments were performed to validate the theoretical calculations. Figure 5a shows the scanning electron microscopy (SEM) images of the synthesized Cu_2_O cubes, confirming that the exposed facets are predominantly {100}. Figure 5b demonstrates the results of methyl orange (MO) photodegradation for 4-FA-, 4-CA-, and 4-BA-decorated Cu_2_O cubes. The results for 4-FA-functionalized octahedra and rhombic dodecahedra are also presented in Figure S11. The extent of MO photodegradation largely increases in the case of Cu_2_O cubes after 4-XA decoration, while only slight enhancement was noted for Cu_2_O octahedra and rhombic dodecahedra. Notably, 4-BA-modified cubes achieve complete MO degradation within 90 min, which aligns well with our DFT results. The moderate enhancements for Cu_2_O {110} and {111} surfaces are also in line with the calculation analysis. To further investigate the impact of surface functionalization on the electronic structure, ultraviolet photoelectron spectroscopy (UPS) was employed. As shown in Figure 6, the experimental work function of Cu_2_O cubes increases from 6.4 to 7.0 eV after 4-XA decoration. While this appears to contradict the work function predicted by DFT, the discrepancy can be rationalized by the proposed mechanism, as shown in Figure 3. Our work function calculations are based on the local vacuum level near the decorators, where the interface effects induced by the halogen substituents lower the local potential barrier, facilitating electron transfer from the surface to the decorator. In contrast, the UPS signal predominantly originates from the underlying Cu_2_O surface and reflects an average over the illuminated region. The net electron withdrawal from Cu_2_O to the molecule, as supported by Bader charge analysis, results in a more positively charged substrate and, thus, a higher experimental work function. This behavior is also observed in RD (Figure S12), whereas octahedra exhibit a negligible change in work function due to their less favorable molecular interaction.

**Figure 5:**
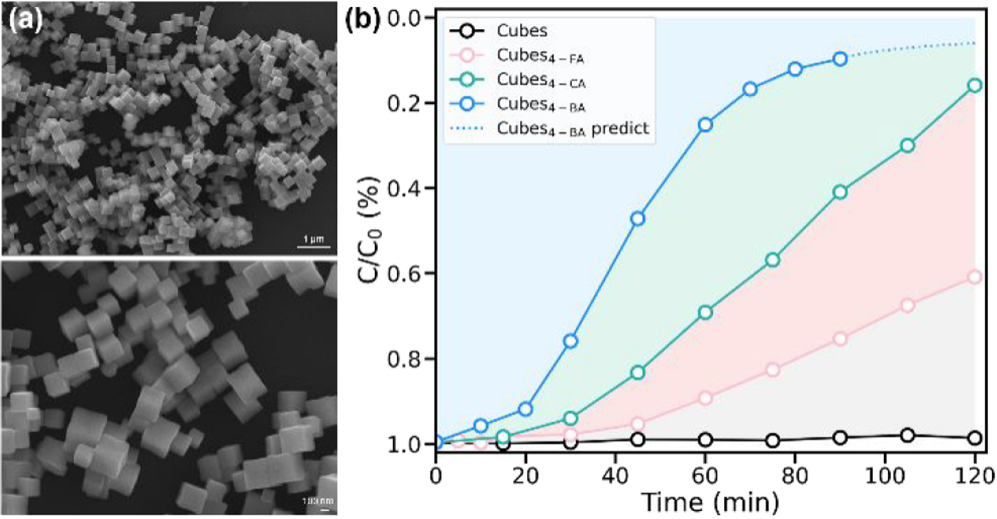
(a) SEM images of the synthesized Cu_2_O cubes. (b) Plots of the extents of methyl orange degradation vs time for pristine- and molecule-modified Cu_2_O cubes.

**Figure 6:**
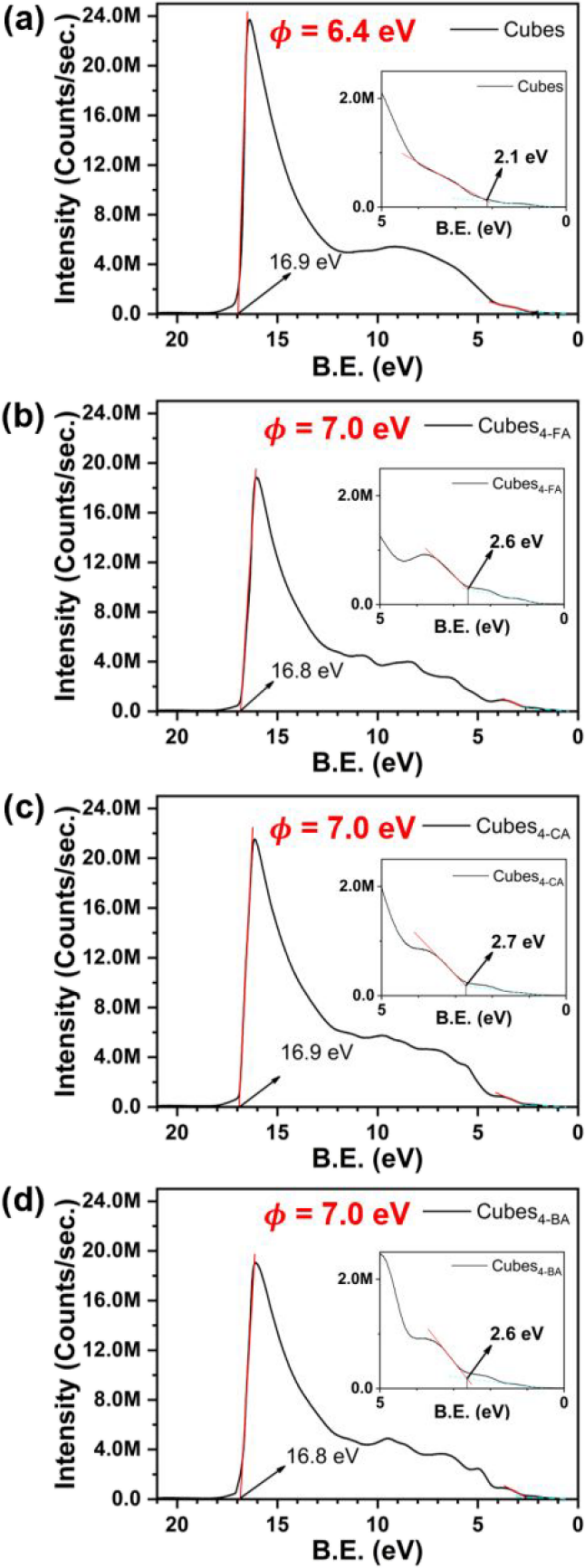
Ultraviolet photoelectron spectra of (a) cubes, (b) 4-FA-modified cubes, (c) 4-CA-modified cubes, and (d) 4-BA-modified cubes.

After a series of studies for molecular functionalization based on Cu_2_O systems,^35–38,41^ our study identifies two crucial factors that significantly influence photocatalytic performance: the presence of a molecule-induced band and the capability of electrons to leave the surface. The significant improvement of photocatalytic activity for the decorated Cu_2_O {100} surface can be attributed to the molecule-induced band within the band gap, large charge separation, decreased value of work function, electron affinity, and ionization energy. On the other hand, for the Cu_2_O {110} surface, although the 4-FA-induced band exists within the band gap, the ability for electrons escaping from the Cu_2_O surface during the photoexcitation process remains nearly the same, which can be explained by the results of the integral value of CDD and the value of electron affinity and ionization energy. Thus, the photocatalytic performance remains almost unchanged in the case of the Cu_2_O {110} surface. As for the Cu_2_O {111} surface, the poor photocatalytic activity can be attributed to the absence of a molecule-induced band, charge localization between the Cu_2_O surface and 4-FA molecule, and the value of work function, electron affinity, and ionization energy remaining nearly the same after decoration. UPS spectra further provide important evidence to the charge transfer, which validates our proposed mechanism.

## 3 Conclusions

In this study, we employed DFT calculations to investigate the charge transfer behavior, electronic structure, and energy band diagrams of Cu_2_O {100}, {110}, and {111} surfaces modified with 4-BA, 4-CA, and 4-FA molecules. Our findings reveal two distinct photocatalytic mechanisms governed by the energy barrier for electrons transferring to the decorating molecules. The calculated results align well with experimental observations, demonstrating that the 4-XA-decorated Cu_2_O {100} surface exhibits significant photocatalytic enhancement, whereas Cu_2_O {110} and {111} surfaces show only moderate improvements. UPS results prove the charge transfer behavior on the Cu_2_O surface. Additionally, we identified two critical factors influencing photocatalytic performance: the presence of molecular states within the band structure and substantial changes in the electron capability from the surface. These insights provide a robust framework for the rational design and optimization of high-performance, eco-friendly photocatalytic systems.

## 4 Method

The Vienna Ab initio simulation package (VASP)^46,47^ was implemented in the first-principles calculations based on DFT with the projector augmented wave^48,49^ method. The Perdew–Burke–Ernzerhof^50^ functional within the generalized-gradient approximations^51^ was conducted for the structure optimization, electronic structure, and the band-decomposed charge density calculations. The cutoff energy for the plane-wave basis set was 460 eV, and the 8 × 8 × 8 Γ-centered *k*-point meshes were utilized to depict Brillouin zone of the Cu_2_O primitive cell. The Cu 3d^10^4s^1^, O 2s^2^2p^4^, H 1s^1^, C 2s^2^2p^2^, F 2s^2^2p^5^, Cl 3s^2^3p^5^, and Br 4s^2^4p^5^ electrons were treated as valence electrons. Gaussian smearing was used, and the smearing width was 0.01 eV. The optimized lattice constant was 4.31 Å, agreeing with the previous studies.^52^ On the other hand, for decorating molecules 4-FA, 4-CA, and 4-BA, a 1 × 1 × 1 *k*-point grid was used. The geometric structure of the Cu_2_O crystal and the decorating molecules was relaxed by using the conjugated-gradient method. We used a 1.0 × 10^–5^ eV energy convergence criterion for both the Cu_2_O crystal and the decorating molecules for electronic relaxation and 1.0 × 10^–4^ eV energy convergence for ionic relaxation.

Subsequently, the {100}, {110}, and {111} surfaces of Cu_2_O crystals were created by using the supercells from the optimized primitive cell. Larger than 18 Å vacuum space was built to separate the periodic boundaries along the surface normal direction, thereby effectively avoiding any unintended interactions existing between adjacent molecules and slabs. Then, the 4-FA-, 4-CA-, and 4-BA-decorated Cu_2_O surfaces were optimized with a *k*-point mesh of 2 × 2 × 1 and a cutoff energy of 460 eV. For the decorated {100} surface, a 4 × 4 supercell was used, while a 3 × 3 supercell was used for the decorated {110} and {111} surfaces. To calculate the binding energy of Cu_2_O-decorated systems, we used the following formula:

$$E_{\mathrm{binding}}=E_{\mathrm{deco}}-E_{{\mathrm{Cu}}_{2}O}-E_{4‐\mathrm{XA}}$$ 1

where *E*_deco_ is the energy of the decorated surface; *E*_Cu_2_O_ and *E*_4‑XA_ are the energy of the pristine Cu_2_O surface and the 4-XA molecule, respectively. Next, to evaluate the charge transfer between the 4-XA molecule and the Cu_2_O surface, the charge density difference was calculated according to the following equation:

$$\Delta \rho =\rho_{\mathrm{deco}}-\rho_{{\mathrm{Cu}}_{2}O}-\rho_{4‐XA}$$ 2

where ρ_deco_ is the charge density of the decorated surface; ρ_Cu_2_O_ and ρ_4‑XA_ are the charge densities of the pristine Cu_2_O surface and the 4-XA molecule, respectively. We also performed the Bader charge difference analysis to quantify the charge over the decorated Cu_2_O surface, which is defined as the Bader charge of the decorated surface minus that of the unmodified one.^53,54^ A positive value indicates a tendency of the atom to acquire electrons. We also present the planar average charge density difference along the z direction and energy diagram of different Cu_2_O surface systems.

## Supplementary Material



## Notes

Cu_2_O crystal synthesis conditions; photodegradation experiment conditions, instrumentation, UPS measurement details, 4-XA-decorated Cu_2_O surface DFT model and the corresponding binding energies; CDD and the planar average electrostatic potential results of the 4-FA- and 4-CA-decorated Cu_2_O {100} surface; calculation results of the 4-FA- and 4-BA-decorated {110} and {111} surface; band diagram; photocatalysis experiments of 4-FA-modified RD and octahedra; and UPS spectra of RD and octahedra (PDF)

## Abbreviations

4-FA: 1-ethynyl-4-fluorobenzene
4-CA: 1-chloro-4-ethynylbenzene
4-BA: 1-bromo-4-ethynylbenzene
RD: rhombic dodecohedra
DFT: density functional theory
4-EA: 4-ethynyl-aniline
4-TFMA: 4-trifluoromethylphenylacetylene
2E-6MN: 2-ethynyl-6-methoxynaphthalene
4-NA: 4-nitrophenylacetylene
4-CNA: 4-cyano-phenylacetylene
MEP: molecular electrostatic potential
ELF: electron localization function
FT-IR: Fourier-transform infrared
CDD: charge density difference
BCD: Bader charge difference
CBM: conduction band minimum
VBM: valence band maximum
MO: methyl orange
UPS: ultraviolet photoelectron spectroscopy
VASP: Vienna Ab initio simulation package

## References

[1] Fresno F., Portela R., Suárez S., Coronado J. M. (2014). Photocatalytic Materials:
Recent Achievements and near Future Trends. J. Mater. Chem. A.

[2] Lee S.-Y., Park S.-J. (2013). TiO_2_ Photocatalyst
for Water Treatment Applications. J. Ind. Eng.
Chem..

[3] Lee K. M., Lai C. W., Ngai K. S., Juan J. C. (2016). Recent
Developments
of Zinc Oxide Based Photocatalyst in Water Treatment Technology: A
Review. Water Res..

[4] Ren H., Koshy P., Chen W.-F., Qi S., Sorrell C. C. (2017). Photocatalytic
Materials and Technologies for Air Purification. J. Hazard. Mater..

[5] Zhang L., Zhang J., Yu H., Yu J. (2022). Emerging S-Scheme
Photocatalyst. Adv. Mater..

[6] Tzirakis M. D., Lykakis I. N., Orfanopoulos M. (2009). Decatungstate
as an Efficient Photocatalyst
in Organic Chemistry. Chem. Soc. Rev..

[7] Tong H., Ouyang S., Bi Y., Umezawa N., Oshikiri M., Ye J. (2012). Nano-photocatalytic
Materials: Possibilities and Challenges. Adv.
Mater..

[8] Li Y., Gao C., Long R., Xiong Y. (2019). Photocatalyst Design Based on Two-Dimensional
Materials. Mater. Today Chem..

[9] Sakthivel S., Neppolian B., Shankar M. V., Arabindoo B., Palanichamy M., Murugesan V. (2003). Solar Photocatalytic Degradation
of Azo Dye: Comparison of Photocatalytic Efficiency of ZnO and TiO_2_. Sol. Energy Mater. Sol. Cells.

[10] Rauf M. A., Ashraf S. S. (2009). Fundamental Principles and Application of Heterogeneous
Photocatalytic Degradation of Dyes in Solution. Chem. Eng. J..

[11] Kudo A., Miseki Y. (2009). Heterogeneous Photocatalyst
Materials for Water Splitting. Chem. Soc. Rev..

[12] Liu J., Liu Y., Liu N., Han Y., Zhang X., Huang H., Lifshitz Y., Lee S.-T., Zhong J., Kang Z. (2015). Metal-Free
Efficient Photocatalyst for Stable Visible Water Splitting via a Two-Electron
Pathway. Science.

[13] Opoku F., Govender K. K., Van Sittert C. G. C.
E., Govender P. P. (2017). Recent
Progress in the Development of Semiconductor-Based Photocatalyst Materials
for Applications in Photocatalytic Water Splitting and Degradation
of Pollutants. Adv. Sustain. Syst..

[14] Ran J., Jaroniec M., Qiao S. (2018). Cocatalysts
in Semiconductor-based
Photocatalytic CO_2_ Reduction: Achievements, Challenges,
and Opportunities. Adv. Mater..

[15] Fu J., Jiang K., Qiu X., Yu J., Liu M. (2020). Product Selectivity
of Photocatalytic CO_2_ Reduction Reactions. Mater. Today.

[16] Lang X., Chen X., Zhao J. (2014). Heterogeneous Visible
Light Photocatalysis
for Selective Organic Transformations. Chem.
Soc. Rev..

[17] Shiraishi Y., Hirai T. (2008). Selective Organic Transformations on Titanium Oxide-Based Photocatalysts. J. Photochem. Photobiol. C: Photochem. Rev..

[18] Majeed I., Manzoor U., Kanodarwala F. K., Nadeem M. A., Hussain E., Ali H., Badshah A., Stride J. A., Nadeem M. A. (2018). Pd–Ag Decorated
g-C_3_N_4_ as an Efficient Photocatalyst for Hydrogen
Production from Water under Direct Solar Light Irradiation. Catal. Sci. Technol..

[19] Shiuan
Ng L., Raja Mogan T., Lee J.-K., Li H., Ken Lee C.-L., Kwee Lee H. (2023). Surface-Degenerate Semiconductor Photocatalysis for
Efficient Water Splitting without Sacrificial Agents via a Reticular
Chemistry Approach. Angew. Chem., Int. Ed..

[20] Chen Y.-A., Nakayasu Y., Lin Y.-C., Kao J.-C., Hsiao K.-C., Le Q.-T., Chang K.-D., Wu M.-C., Chou J.-P., Pao C.-W., Chang T.-F. M., Sone M., Chen C.-Y., Lo Y.-C., Lin Y.-G., Yamakata A., Hsu Y.-J. (2024). Double-Hollow
Au@CdS Yolk@Shell Nanostructures as Superior Plasmonic Photocatalysts
for Solar Hydrogen Production. Adv. Funct. Mater..

[21] Chen Y.-C., Huang X.-F., Hsu H.-T., Wu E.-T., Peng C.-H., Huang M. H. (2024). Photocatalyzed Dimethylacrylamide
Polymerization in
an Aqueous Solution Using 4-Nitrophenylacetylene-Modified Cu_2_O Crystals. J. Mater. Chem. A.

[22] Wang G.-R., Huang M. H. (2024). Photocatalytic Oxidative
Cyclization of Aromatic Thioamides
Catalyzed by Cu_2_O Rhombic Dodecahedra. J. Mater. Chem. A.

[23] Yuan G.-Z., Hsia C.-F., Lin Z.-W., Chiang C., Chiang Y.-W., Huang M. H. (2016). Highly Facet-Dependent
Photocatalytic Properties of
Cu_2_O Crystals Established through the Formation of Au-Decorated
Cu_2_O Heterostructures. Chem.—Eur.
J..

[24] Majumder D., Chakraborty I., Mandal K., Roy S. (2019). Facet-Dependent Photodegradation
of Methylene Blue Using Pristine CeO_2_ Nanostructures. ACS Omega.

[25] Chen Y.-J., Chiang Y.-W., Huang M. H. (2016). Synthesis
of Diverse Ag_2_O Crystals and Their Facet-Dependent Photocatalytic
Activity Examination. ACS Appl. Mater. Interfaces.

[26] Hsieh M.-S., Su H.-J., Hsieh P.-L., Chiang Y.-W., Huang M. H. (2017). Synthesis
of Ag_3_PO_4_ Crystals with Tunable Shapes for Facet-Dependent
Optical Property, Photocatalytic Activity, and Electrical Conductivity
Examinations. ACS Appl. Mater. Interfaces.

[27] Hsieh P.-L., Naresh G., Huang Y.-S., Tsao C.-W., Hsu Y.-J., Chen L.-J., Huang M. H. (2019). Shape-Tunable SrTiO_3_ Crystals
Revealing Facet-Dependent Optical and Photocatalytic Properties. J. Phys. Chem. C.

[28] Kao J.-C., Chou J.-P., Chen P.-J., Lo Y.-C. (2023). Using Adatoms to
Tune the Band Structures of Cu_2_O Surfaces for Photocatalytic
Applications. Mater. Today Phys..

[29] Pu Y.-C., Chou H.-Y., Kuo W.-S., Wei K.-H., Hsu Y.-J. (2017). Interfacial
Charge Carrier Dynamics of Cuprous Oxide-Reduced Graphene Oxide (Cu_2_O-rGO) Nanoheterostructures and Their Related Visible-Light-Driven
Photocatalysis. Appl. Catal. B: Environ..

[30] Li X., Wei D., Ye L., Li Z. (2019). Fabrication of Cu_2_O-RGO/BiVO_4_ Nanocomposite
for Simultaneous Photocatalytic CO_2_ Reduction and Benzyl
Alcohol Oxidation under Visible Light. Inorg.
Chem. Commun..

[31] Liang T.-Y., Chan S.-J., Patra A. S., Hsieh P.-L., Chen Y.-A., Ma H.-H., Huang M. H. (2021). Inactive Cu_2_O Cubes Become
Highly Photocatalytically Active with Ag_2_S Deposition. ACS Appl. Mater. Interfaces.

[32] Niu P., Dai J., Zhi X., Xia Z., Wang S., Li L. (2021). Photocatalytic
Overall Water Splitting by Graphitic Carbon Nitride. InfoMat.

[33] Ling G. Z. S., Ng S., Ong W. (2022). Tailor-Engineered 2D Cocatalysts:
Harnessing Electron–Hole Redox Center of 2D g-C_3_N_4_ Photocatalysts toward Solar-to-Chemical Conversion
and Environmental Purification. Adv. Funct Materials.

[34] Luo J., Zhang W., Yang H., Fan Q., Xiong F., Liu S., Li D., Liu B. (2021). Halide Perovskite
Composites for
Photocatalysis: A Mini Review. EcoMat.

[35] Chen T.-N., Kao J.-C., Zhong X.-Y., Chan S.-J., Patra A. S., Lo Y.-C., Huang M. H. (2020). Facet-Specific
Photocatalytic Activity
Enhancement of Cu_2_O Polyhedra Functionalized with 4-Ethynylanaline
Resulting from Band Structure Tuning. ACS Cent.
Sci..

[36] Chien K.-C., Yu W.-Y., Kao J.-C., Lo Y.-C., Chou J.-P., Huang M. H. (2024). Photocatalytic Activity Enhancement
with 4-Trifluoromethylphenylacetylene-Functionalized
Cu_2_O Cubes and Rhombic Dodecahedra from Band Structure
Modulation and Use in Boronic Acid Hydroxylation. J. Mater. Chem. A.

[37] Patra A. S., Kao J.-C., Chan S.-J., Chou P.-J., Chou J.-P., Lo Y.-C., Huang M. H. (2022). Photocatalytic
Activity Enhancement
of Cu_2_O Cubes Functionalized with 2-Ethynyl-6-Methoxynaphthalene
through Band Structure Modulation. J. Mater.
Chem. C.

[38] Chan S.-J., Kao J.-C., Chou P.-J., Lo Y.-C., Chou J.-P., Huang M. H. (2022). 4-Nitrophenylacetylene-Modified Cu_2_O Cubes
and Rhombic Dodecahedra Showing Superior Photocatalytic Activity through
Surface Band Structure Modulation. J. Mater.
Chem. C.

[39] Hsieh M.-H., Su Z.-H., Wu E.-T., Huang M. H. (2023). Photocatalytic Aryl
Sulfide Oxidation Using 4-Nitrophenylacetylene-Modified Cu_2_O Crystals. ACS Appl. Mater. Interfaces.

[40] Wu E.-T., Huang M. H. (2023). Photocatalytic
Oxidative Amine Coupling with 4-Nitrophenylacetylene-Modified
Cu_2_O Polyhedra. ACS Catal..

[41] Chou P.-J., Yu W.-Y., Kao J.-C., Lo Y.-C., Chou J.-P., Huang M. H. (2023). Photocatalytic Activity Enhancement with 4-Cyanophenylacetylene-Modified
Cu_2_O Cubes and Rhombic Dodecahedra and Use in Arylboronic
Acid Hydroxylation. J. Mater. Chem. A.

[42] Johnson J.
A., Luo J., Zhang X., Chen Y.-S., Morton M. D., Echeverría E., Torres F. E., Zhang J. (2015). Porphyrin-Metalation-Mediated Tuning
of Photoredox Catalytic Properties in Metal–Organic Frameworks. ACS Catal..

[43] Toyao T., Ueno N., Miyahara K., Matsui Y., Kim T.-H., Horiuchi Y., Ikeda H., Matsuoka M. (2015). Visible-Light, Photoredox
Catalyzed, Oxidative Hydroxylation of Arylboronic Acids Using a Metal–Organic
Framework Containing Tetrakis­(Carboxyphenyl)­Porphyrin Groups. Chem. Commun..

[44] Wei P.-F., Qi M.-Z., Wang Z.-P., Ding S.-Y., Yu W., Liu Q., Wang L.-K., Wang H.-Z., An W.-K., Wang W. (2018). Benzoxazole-Linked
Ultrastable Covalent Organic Frameworks for Photocatalysis. J. Am. Chem. Soc..

[45] Torras-Rosell A., Johannsen S. R., Dirscherl K., Davidsdóttir S., Jeppesen C. S., Louring S., Andersen I. H. (2017). Comparing the Photocatalytic
Activity of TiO_2_ at Macro- and Microscopic Scales. Environ. Sci. Pollut. Res..

[46] Kresse G., Furthmüller J. (1996). Efficiency
of Ab-Initio Total Energy Calculations for
Metals and Semiconductors Using a Plane-Wave Basis Set. Comput. Mater. Sci..

[47] Kresse G., Furthmüller J. (1996). Efficient
Iterative Schemes for Ab Initio Total-Energy
Calculations Using a Plane-Wave Basis Set. Phys.
Rev. B.

[48] Blöchl P. E., Jepsen O., Andersen O. K. (1994). Improved Tetrahedron
Method for Brillouin-Zone
Integrations. Phys. Rev. B.

[49] Kresse G., Joubert D. (1999). From Ultrasoft Pseudopotentials
to the Projector Augmented-Wave
Method. Phys. Rev. B.

[50] Perdew J. P., Burke K., Wang Y. (1996). Generalized
Gradient Approximation
for the Exchange-Correlation Hole of a Many-Electron System. Phys. Rev. B.

[51] Perdew J. P., Burke K., Ernzerhof M. (1996). Generalized
Gradient Approximation
Made Simple. Phys. Rev. Lett..

[52] Ruiz E., Alvarez S., Alemany P., Evarestov R. A. (1997). Electronic
Structure and Properties of Cu_2_O. Phys. Rev. B.

[53] Sanville E., Kenny S. D., Smith R., Henkelman G. (2007). Improved Grid-Based
Algorithm for Bader Charge Allocation. J. Comput.
Chem..

[54] Tang W., Sanville E., Henkelman G. (2009). A Grid-Based Bader Analysis Algorithm
without Lattice Bias. J. Phys.: Condens. Matter.

