# Supplementary material for: Enhanced Photocatalytic Performance of Halogenated Phenylacetylene-Decorated Cu2O Surfaces via Electronic Structure Modulation: A DFT and Experimental Study
Source: ACS Nanosci Au. 2025 Jun 5;5(4):314–23. doi: 10.1021/acsnanoscienceau.5c00030 (PMC12371584; doi:10.1021/acsnanoscienceau.5c00030)
Supplement: Supplementary file 1 [file ng5c00030_si_001.pdf]

Supporting information

Enhanced Photocatalytic Performance of Halogenated Phenylacetylene-Decorated Cu_2O Surfaces via Electronic Structure Modulation: A DFT and Experimental Study

Jui-Cheng Kao[a,‡], Wei-Yang Yu[a,‡], Kuo-Chang Chien[b], Po-Jung Chou[b], Michael H. Huang[b,*], Yu-Chieh Lo[a,*], Jyh-Pin Chou[c,*]

[a] *Department of Materials Science and Engineering, National Yang Ming Chiao Tung University, Hsinchu 30010, Taiwan*
[b] *Department of Chemistry, Frontier Research Center on Fundamental and Applied Sciences of Matters, National Tsing Hua University, Hsinchu 30013, Taiwan*
[c] *Graduate School of Advanced Technology National Taiwan University, Taipei 106319, Taiwan.*

[‡]The author contributed equally.
*Corresponding Author(s): Michael H. Huang (hyhuang@mx.nthu.edu.tw); Yu-Chieh Lo (yclo@nycu.edu.tw); Jyh-Pin Chou (jpchou@ntu.edu.tw)

Chemicals

Copper(II) chloride anhydrous ($CuCl_2$, 98%, Alfa Aesar), hydroxylamine hydrochloride ($NH_2OH·HCl$, 99%, Sigma–Aldrich), sodium hydroxide (NaOH, 98%, SHOWA), sodium dodecyl sulfate ($C_{12}H_{25}NaSO_4$, ≥99.0%, J. T. Baker), absolute ethanol (C_2H_5OH, ≥99.5%, Honeywell), 1-ethynyl-4-fluorobenzene (C_8H_5F, 99%, Alfa Aesar), 1-chloro-4-ethynylbenzene (C_8H_5Cl, 98%, Combi-Blocks), 1-bromo-4-ethynylbenzene (C_8H_5Br, 98%, TCI), methyl orange ($C_{14}H_{14}N_3NaO_3S$, Alfa Aesar), potassium carbonate (K_2CO_3, 99%, Alfa Aesar), sodium oxalate ($Na_2C_2O_4$, ≥99.0%, Sigma–Aldrich), and potassium bromate ($KBrO_3$, 99%, Alfa Aesar) were used as received. A spin-trapping reagent 5,5-dimethyl-1-pyrroline N-oxide ($C_6H_{11}NO$,> 97.0 %, TCI) was purified with activated carbon. Milli-Q water (18.2 MΩ) was used to prepare all the solutions in the experiments.

Synthesis of Cu_2O Crystals.

All Cu_2O crystals were synthesized in a 31 ºC water bath. To grow Cu_2O cubes, 114.6 mL of deionized water was added to a vial containing 1.044 g of SDS. Next, 1.2 mL of 0.1 M $CuCl_2$ solution was added with stirring for 25 min. After that, 2.4 mL of 1 M NaOH solution was introduced and stirred for 5 sec. Finally, 1.8 mL of 0.2 M $NH_2OH·HCl$ solution was quickly added and stirred for 10 sec. After stop stirring, the solution was aged for 50 min.

To prepare Cu_2O octahedra, 26.26 mL of deionized water was added to a sample vial containing 0.348 g of SDS, followed by the addition of 0.8 mL of 0.1 M $CuCl_2$ solution with stirring for 25 min. After that, 0.8 mL of 1 M NaOH was introduced and stirred for 3 sec. Finally, 2.6 mL of 0.2 M $NH_2OH·HCl$ solution was quickly added and stirred for 10 sec. After stop stirring, the solution was aged for 25 min.

To synthesize Cu_2O rhombic dodecahedra, 27.68 mL of deionized water was added to a sample vial containing 0.348 g of SDS. Then 2 mL of 0.1 M $CuCl_2$ solution was added with stirring for 25 min. After that, 0.72 mL of 1 M NaOH was added and stirred for 5 sec, then 9.6 mL of 0.1 M $NH_2OH·HCl$ was quickly added and stirred for 20 sec. After stop stirring, the solution was aged for 50 min.

The solid product was centrifuged at 7500 rpm for 3 min, and washed with 1:1 volume ratio of water and ethanol for three times to remove SDS and residual chemicals, and then washed with 95% ethanol once. After washing, the particles were kept in absolute ethanol to avoid oxidation.

Photodegradation Experiment.

The weights of 4-XA-functionalized Cu_2O cubes (7.9 mg), rhombic dodecahedra (8.4 mg), and octahedra (4.0 mg) having a total particle surface area of 0.03 m^2 were used. Cu_2O crystals were added to a 4 cm × 4 cm × 4 cm quartz cell, and the cell was filled up to 45 mL with 15 ppm of a methyl orange solution. The quartz cell was placed 30 cm away from a xenon lamp with a Y-43 cutoff filter used to block light below 400 nm. The measured power density reaching the cell was about 1.00 W/cm^2. During light illumination, 1 mL of the solution was withdrawn from the cell at certain time intervals and centrifuged to remove Cu_2O particles. The MO solution was recorded for UV−vis spectra.

Instrumentation.

SEM images were taken using a JEOL JSM-7000F electron microscope. The ultraviolet photoelectron spectroscopy (UPS) measurement were taken using a AES/ESCA scanning microprobe (ULVAC-PHI PHI 5000 Versaprobe III).

UPS measurement.

Sample preparation:
2.5 mg of Cu_2O particle powder was added to 0.5 mL of ethanol and sonicated, then loaded the solution by drops to a 1.5 cm × 1.0 cm ITO glass until the coverage was even. The substrate was placed in a vacuum oven overnight before UPS measurement.

4-XA binding behavior on Cu_2O surfaces.

Table S1 shows the binding energies comparison between the molecular plane placed parallel and vertical to the surface of the 4-XA molecules on the Cu_2O {100}, {110}, and {111} surfaces. The corresponding geometric configurations are shown in **Table S2-S4**. For the {100} surface, the binding energy of the vertical configuration is smaller than that of the parallel-1 configuration among all the 4-XA molecules. Such results suggest the much more stable configuration (i.e. vertical). For the parallel-2 configuration, except for 4-CA, the similar binding energies can be attributed to the quasi- surface reconstruction from the surface atom and the formation of the C-Cu bonding. After the structure relaxation, the 4-XA molecule no longer lies completely flat on the surface but tends to bond in a more vertical orientation. As for the {110} and {111} surfaces, the binding energies of the vertical configuration are more negative compared to those of parallel configurations. Therefore, the 4-XA molecules tend to bind along the surface normal direction. It is worth mentioning that in the binding configurations of these three surfaces, the formation of bonds between C and surface Cu atoms was observed. This result suggests the presence of metal cation–π interactions.

Table S1. The binding energies in the unit of eV of the 4-XA on Cu_2O {100}, {110}, and {111} surfaces.

	**{100}**			**{110}**		**{111}**	
	4-BA	4-CA	4-FA	4-BA	4-FA	4-BA	4-FA
**Parallel-1**	−3.74	−3.78	−2.30	−0.04	−0.05	−1.01	−1.49
**Parallel-2**	−3.93	−5.16	−3.99	−0.11	−0.11	−0.77	−0.77
**Vertical**	−3.95	−3.98	−3.95	−2.16	−2.16	−4.29	−4.32

Table S2. The top and side views of the parallel and vertical geometric configurations of the 4-XA on Cu_2O {100} surface.

	**4-BA**	**4-CA**	**4-FA**
**Parallel-1**	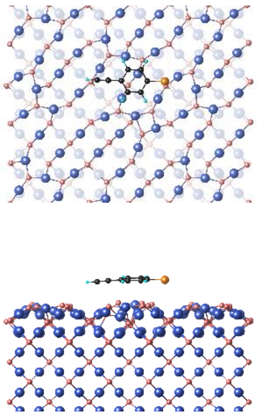	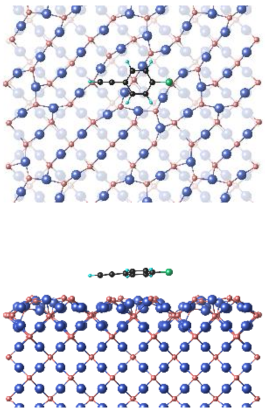	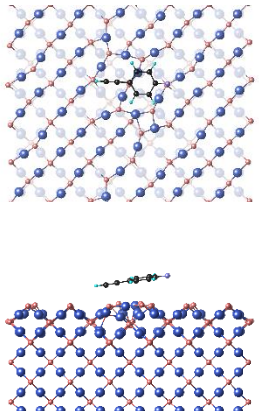
**Parallel-2**	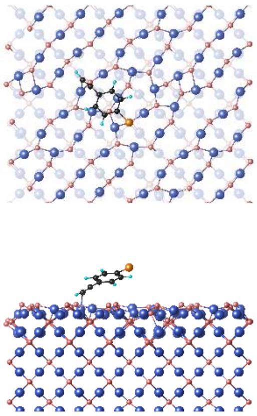	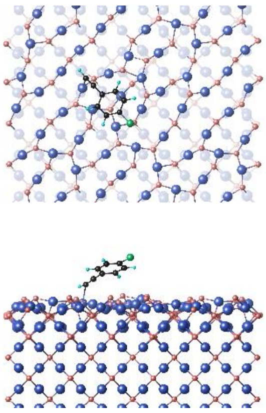	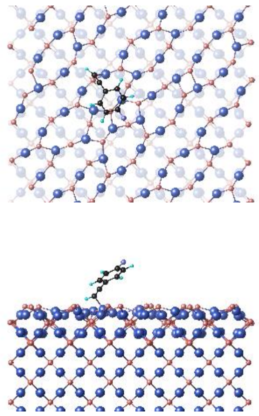
**Vertical**	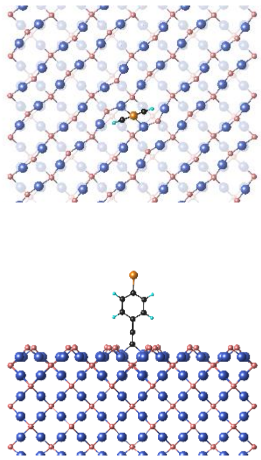	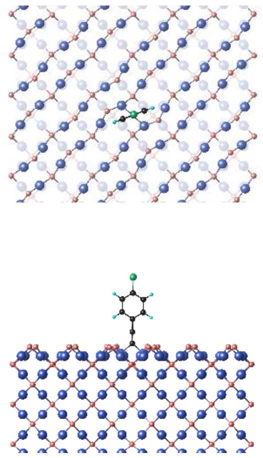	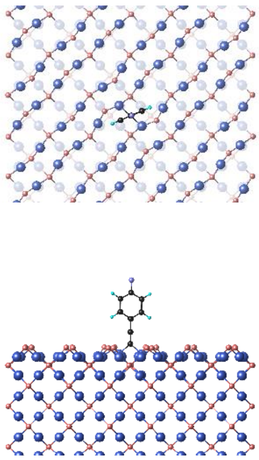

Table S3. The top and side views of the parallel and vertical geometric configurations of the 4-XA on Cu_2O {110} surface.

	**4-BA**	**4-FA**
**Parallel-1**	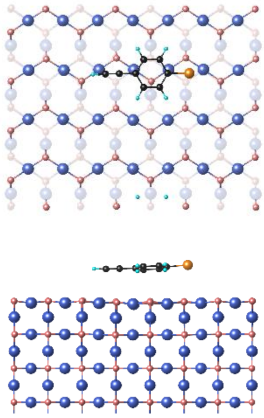	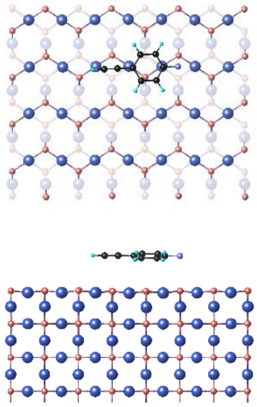
**Parallel-2**	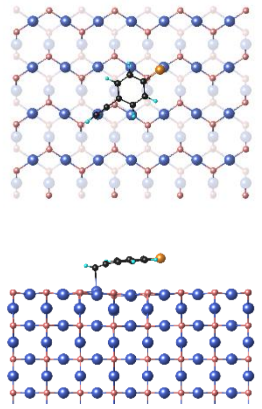	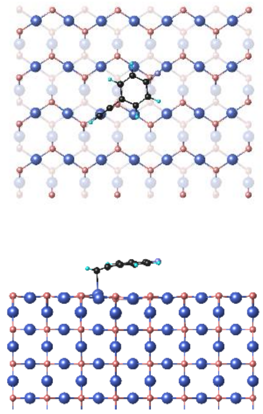
**Vertical**	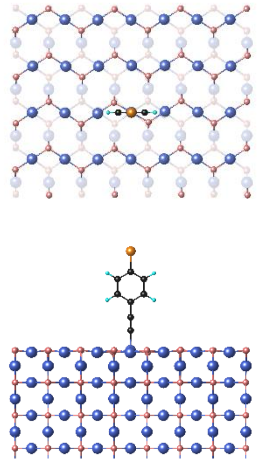	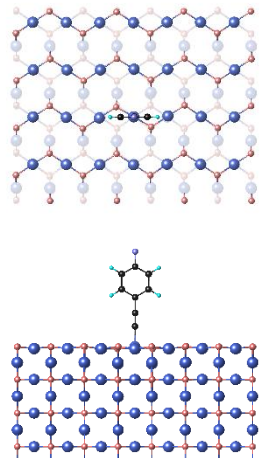

Table S4. The top and side views of the parallel and vertical geometric configurations of the 4-XA on Cu_2O {111} surface.

	**4-BA**	**4-FA**
**Parallel-1**	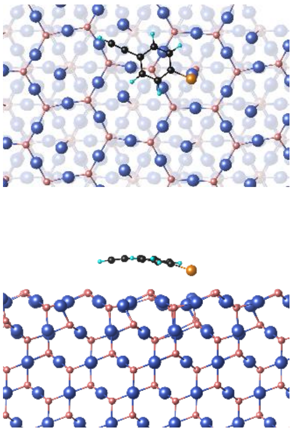	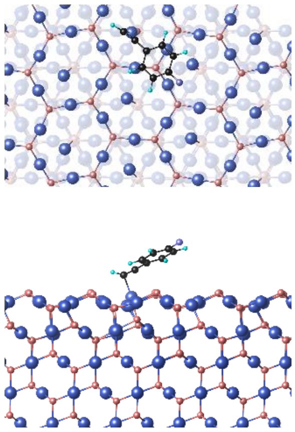
**Parallel-2**	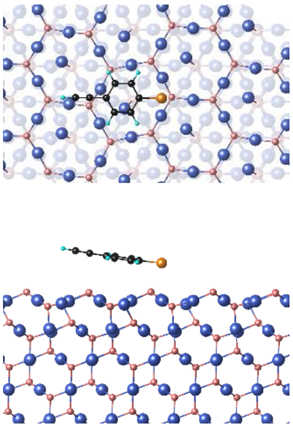	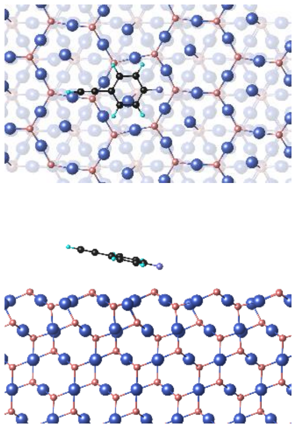
**Vertical**	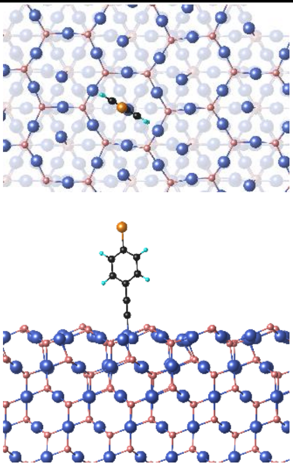	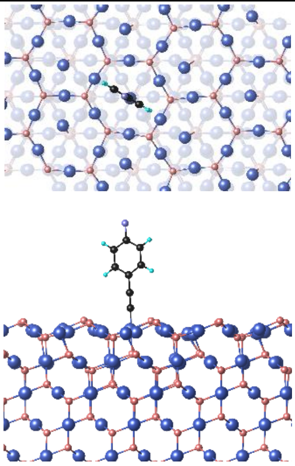

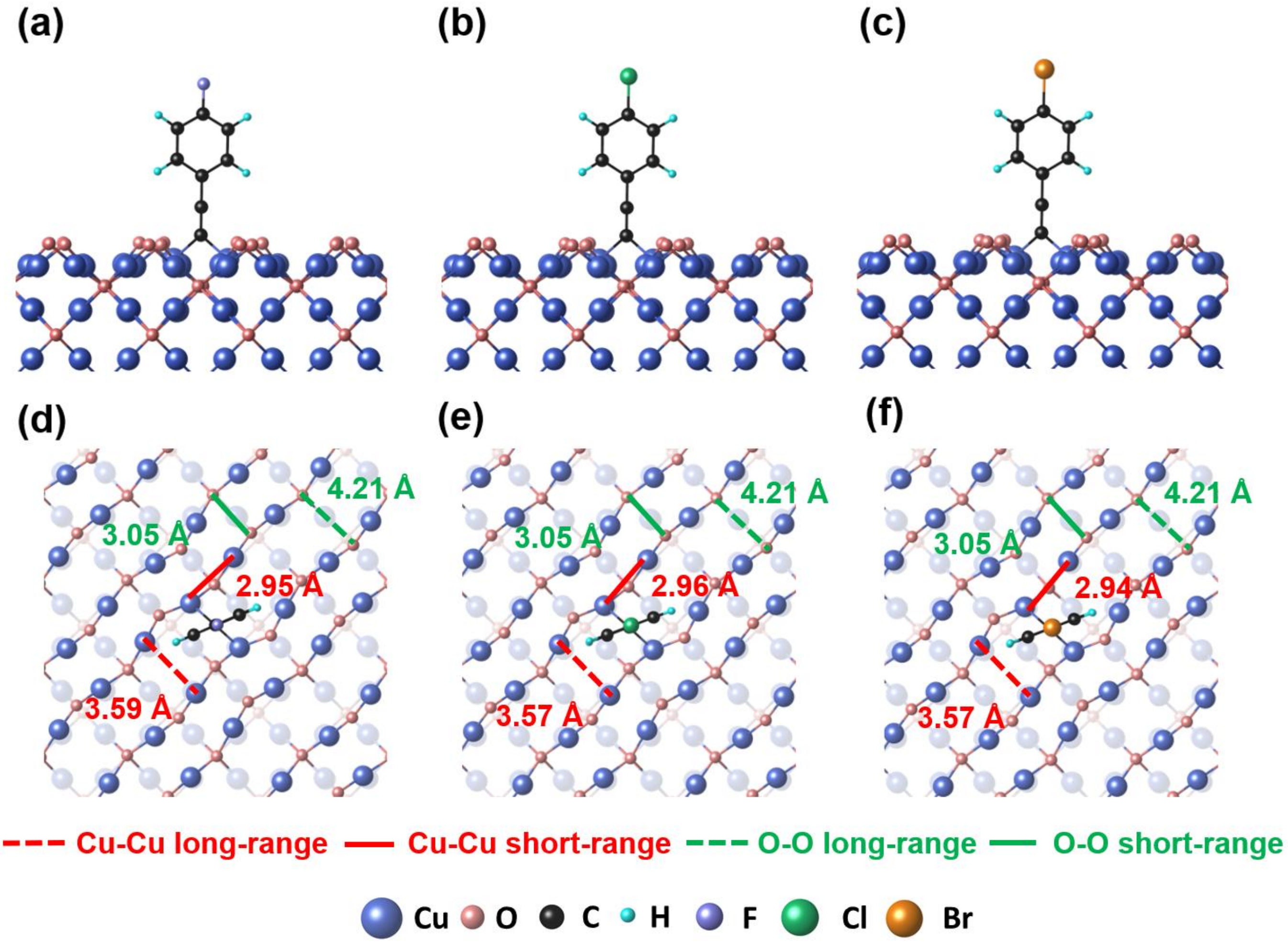

Figure S1. Side view of (a) 4-FA, (b) 4-CA, and (c) 4-BA decorated Cu_2O {100} surfaces. (d-f) The corresponding top view of (a-c).

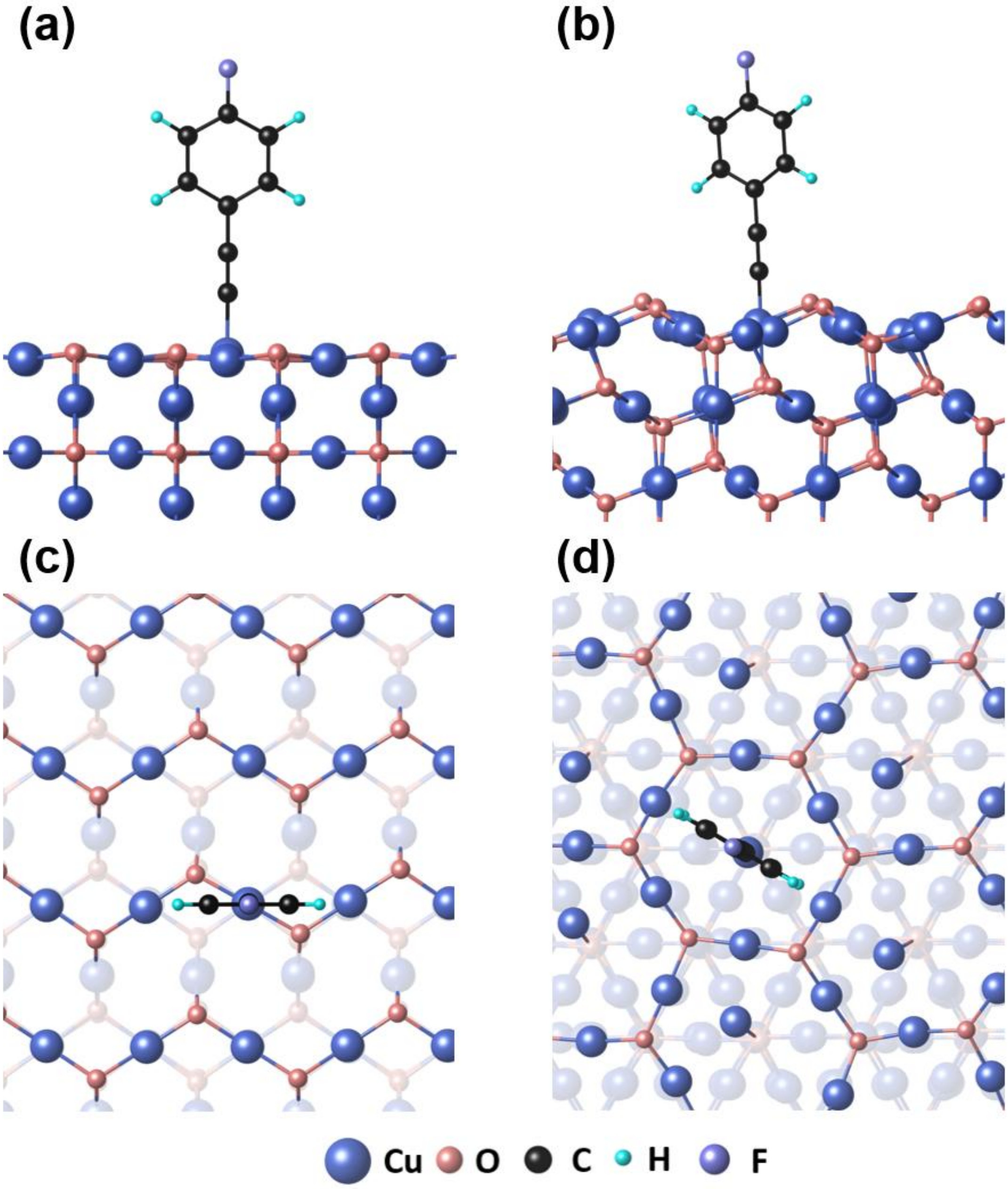

Figure S2. Side view of 4-FA decorated Cu_2O (a) {110} and (b) {111} surfaces. (c, d) The corresponding top view of (a, b).

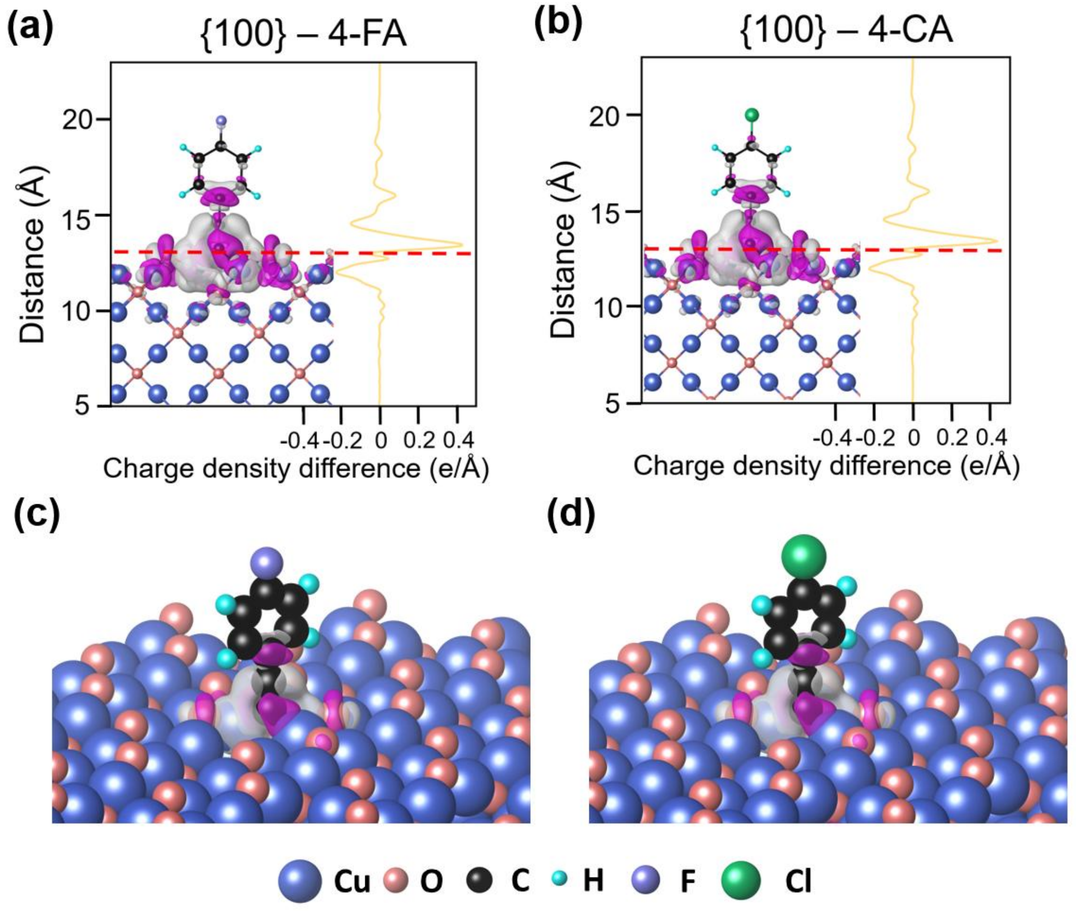

Figure S3. Planar average charge density difference (CDD) along the surface normal for the Cu_2O {100} surface decorated with (a) 4-FA and (b) 4-CA molecules and the side view in the 3D representations. (c, d) The oblique view of CDD for the Cu_2O {100} surface decorated with 4-FA and 4-CA molecules, respectively. The isosurface value is set to 0.001 e/$Bohr^3$. The purple and gray regions represent the charge accumulation and depletion, respectively.

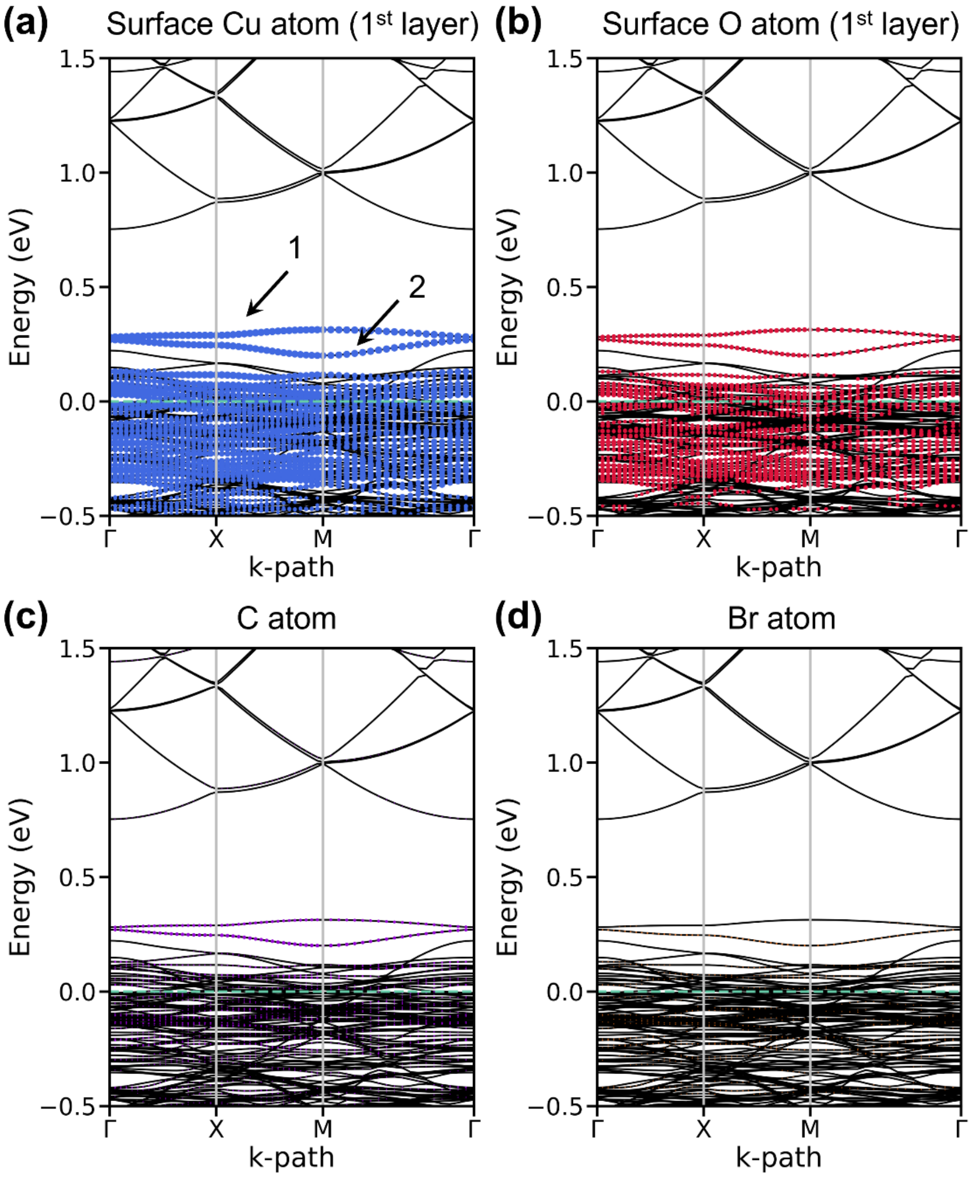

Figure S4. The projected band structures of the Cu_2O {100} surface decorated by 4-BA. The projection of (a) 1st surface Cu atoms, (b) 1st surface O atoms, (c) C atoms, and (d) Br atoms.

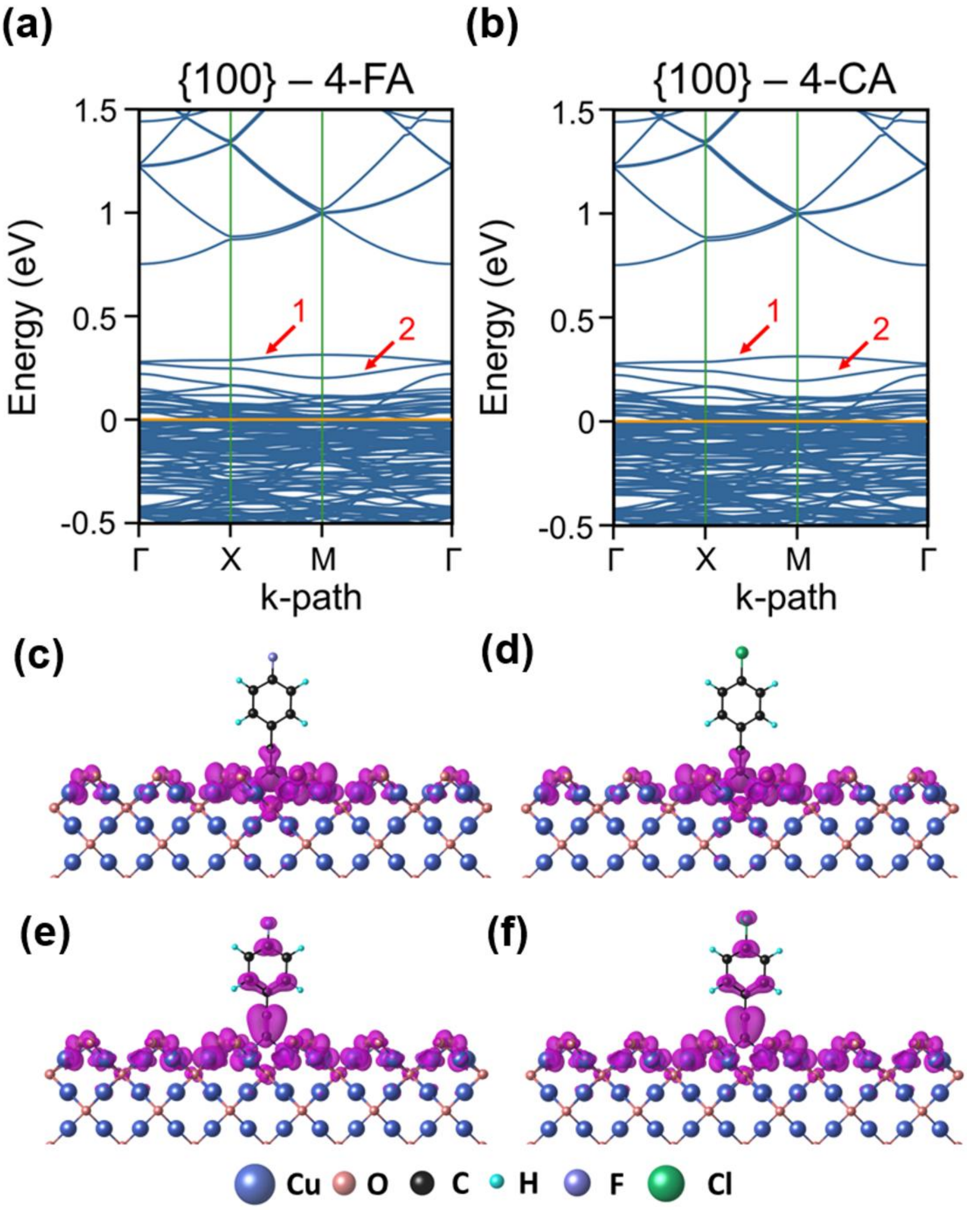

Figure S5. Band structure of Cu_2O {100} surface decorated with (a) 4-FA and (b) 4-CA molecules. The band-decomposed charge density of the (c, d) higher (denoted as 1) and (e, f) lower (denoted as 2) 4-FA and 4-CA-induced bands within the band gap for the {100} surface. The orange lines are Fermi levels, which are all aligned to zero.

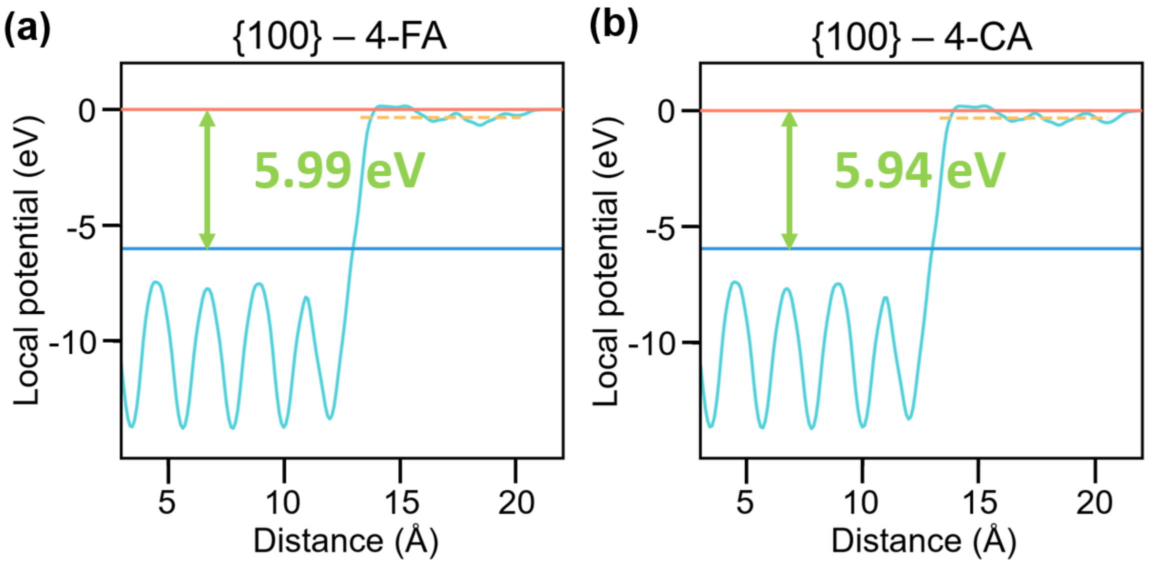

Figure S6. The planar average electrostatic potential along the surface normal for Cu_2O {100} surface decorated with (a) 4-FA and (b) 4-CA molecules. The red and blue solid lines are denoted as vacuum level and Fermi level, respectively. The yellow dashed line represents the average potential of the decorating molecule.

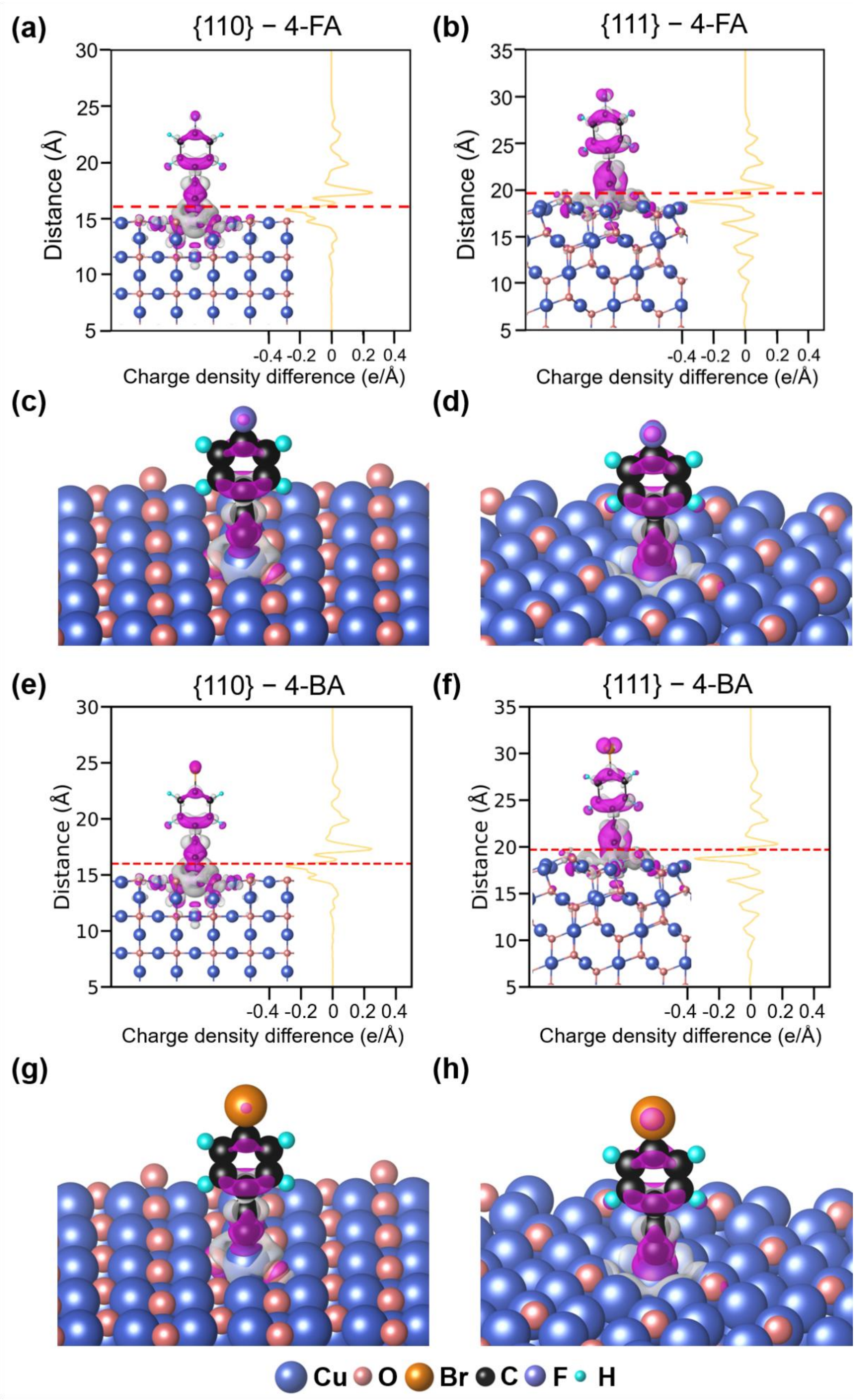

Figure S7. Planar average charge density difference (CDD) along the surface normal for the Cu_2O (a) {110} and (b) {111} surfaces decorated with 4-FA molecules and the side view in the 3D representations. (c-d) The corresponding oblique view of CDD for the decorated Cu_2O {110} and {111} surfaces. Planar average CDD for the Cu_2O (e) {110} and (f) {111} surfaces decorated with 4-BA molecules and the side view in the 3D representations. (g-h) The corresponding oblique view of CDD for the decorated Cu_2O {110} and {111} surfaces. The isosurface value is set to 0.001 e/Bohr^3. The purple and gray regions represent the charge accumulation and depletion, respectively.

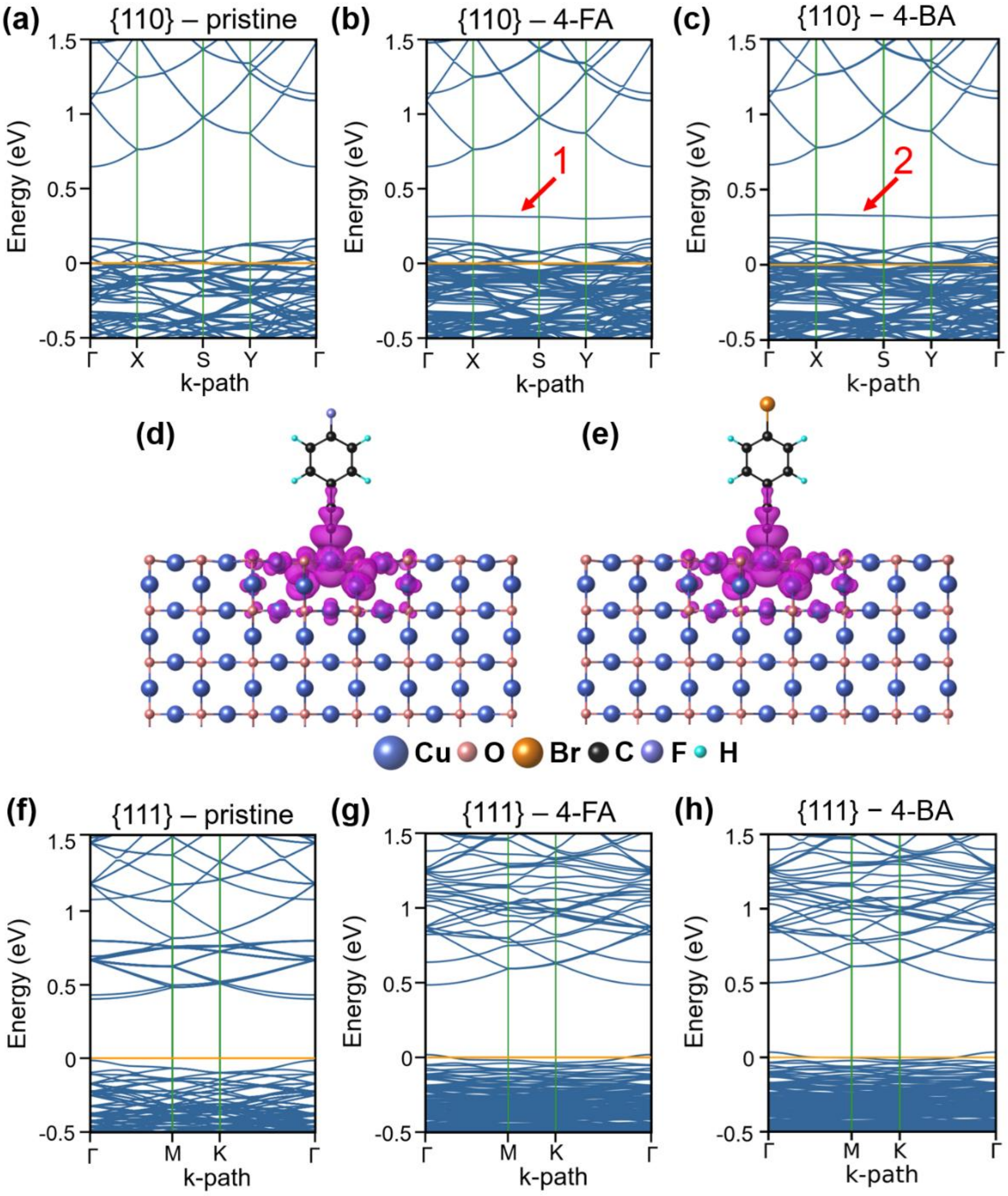

Figure S8. Band structures of Cu_2O {110} and {111} surfaces (a, f) before and after being decorated with (b, g) 4-FA and (c, h) 4-BA molecule. The band-decomposed charge density of the (d) 4-FA-induced bands (denoted as 1) and (e) 4-BA-induced bands (denoted as 2) within the band gap for {110} surface. The orange lines in the band structures are denoted as Fermi level, which are all aligned to zero. The isosurface value is set to 0.001 e/Bohr^3.

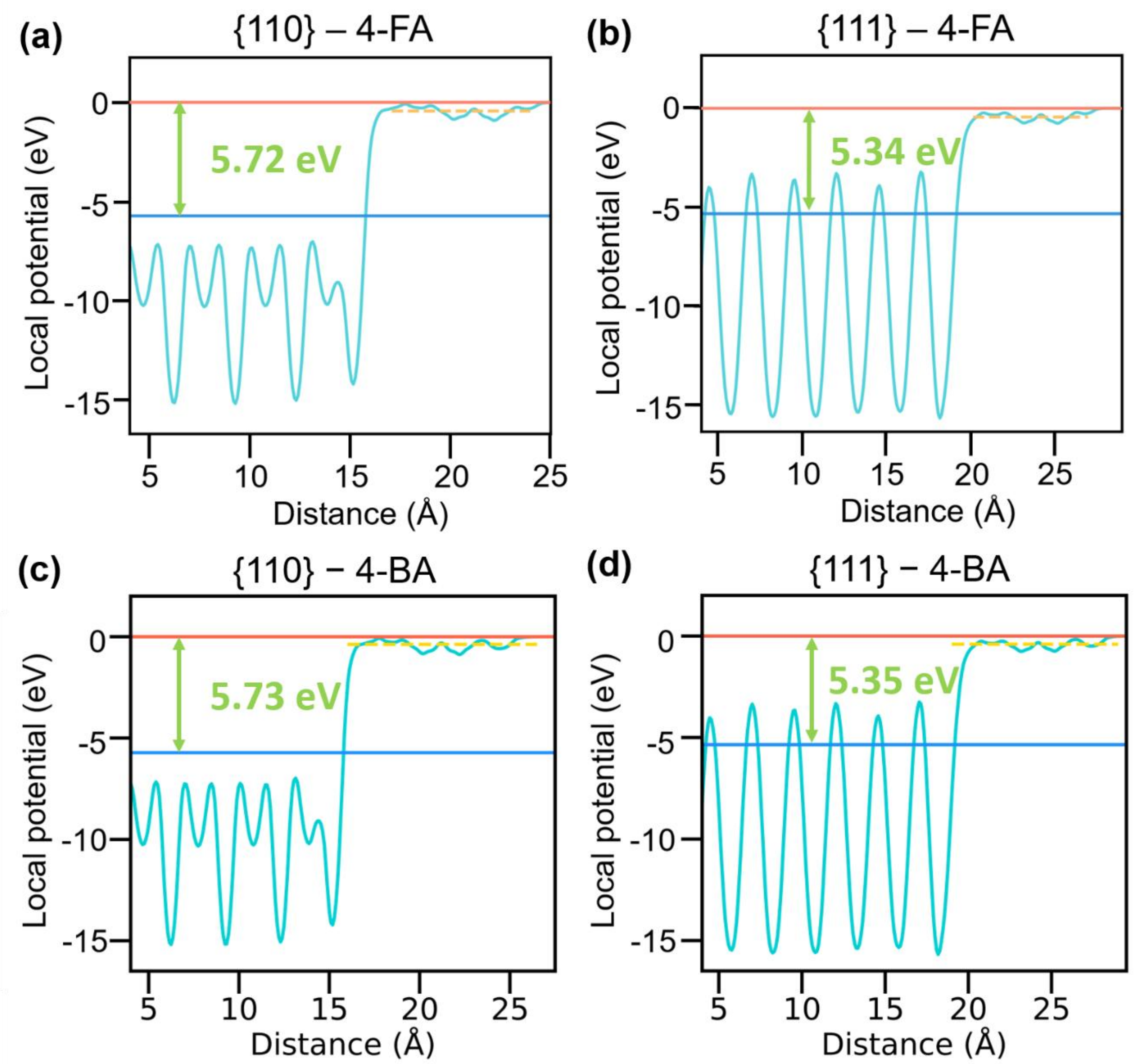

Figure S9. The planar average local potential along the surface normal for Cu_2O 4-FA decorated (a) {110} and (b) {111} surfaces, and 4-BA decorated (c) {110} and (d) {111} surfaces. The red and blue solid lines are denoted as vacuum and Fermi level, respectively. The yellow dashed line represents the average potential of the 4-FA and 4-BA molecules.

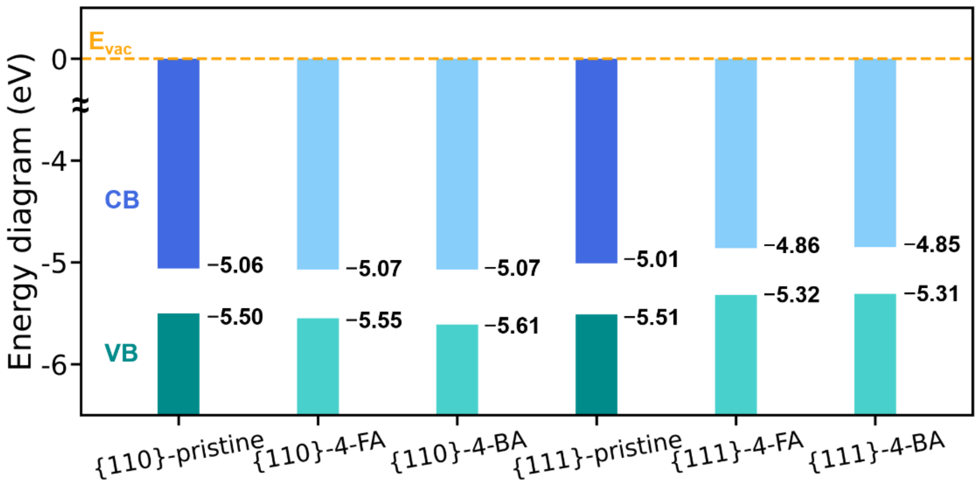

Figure S10. Band diagram for Cu_2O {110} and {111} surfaces before and after 4-FA and 4-BA decoration. E_{vac} stands for the energy of the vacuum level and is aligned to zero (orange dashed line). The upper and lower values represent CBM and VBM, respectively.

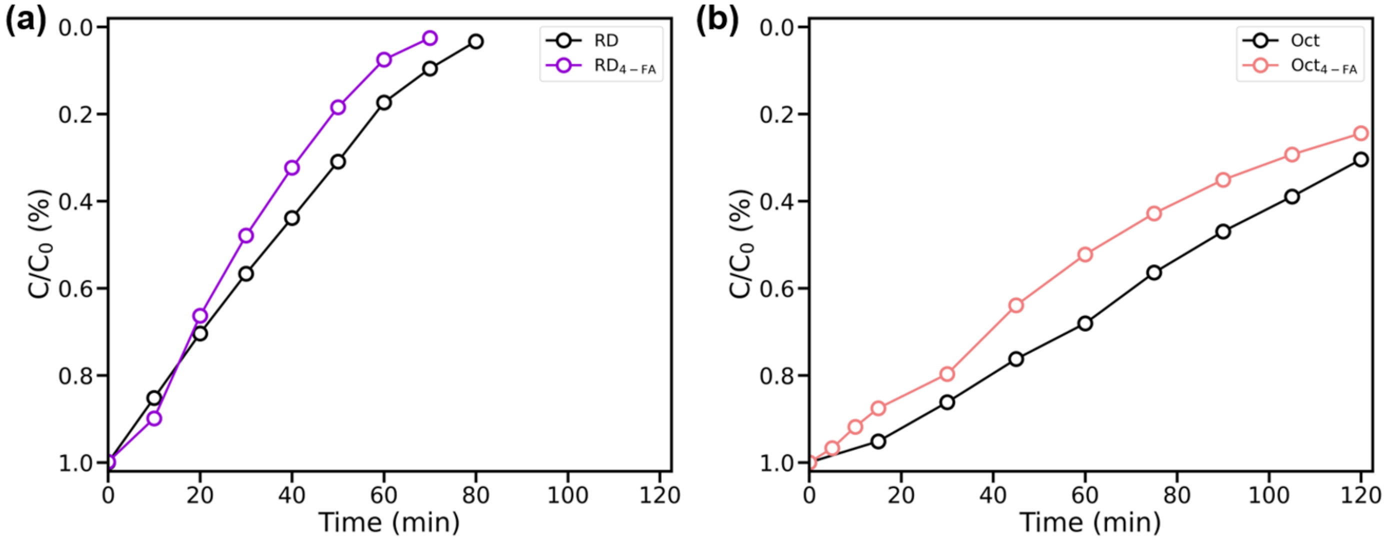

Figure S11. Plots of the extents of methyl orange degradation vs. time for pristine and 4-FA-modified Cu_2O (a) rhombic dodecahedra and (b) octahedra.

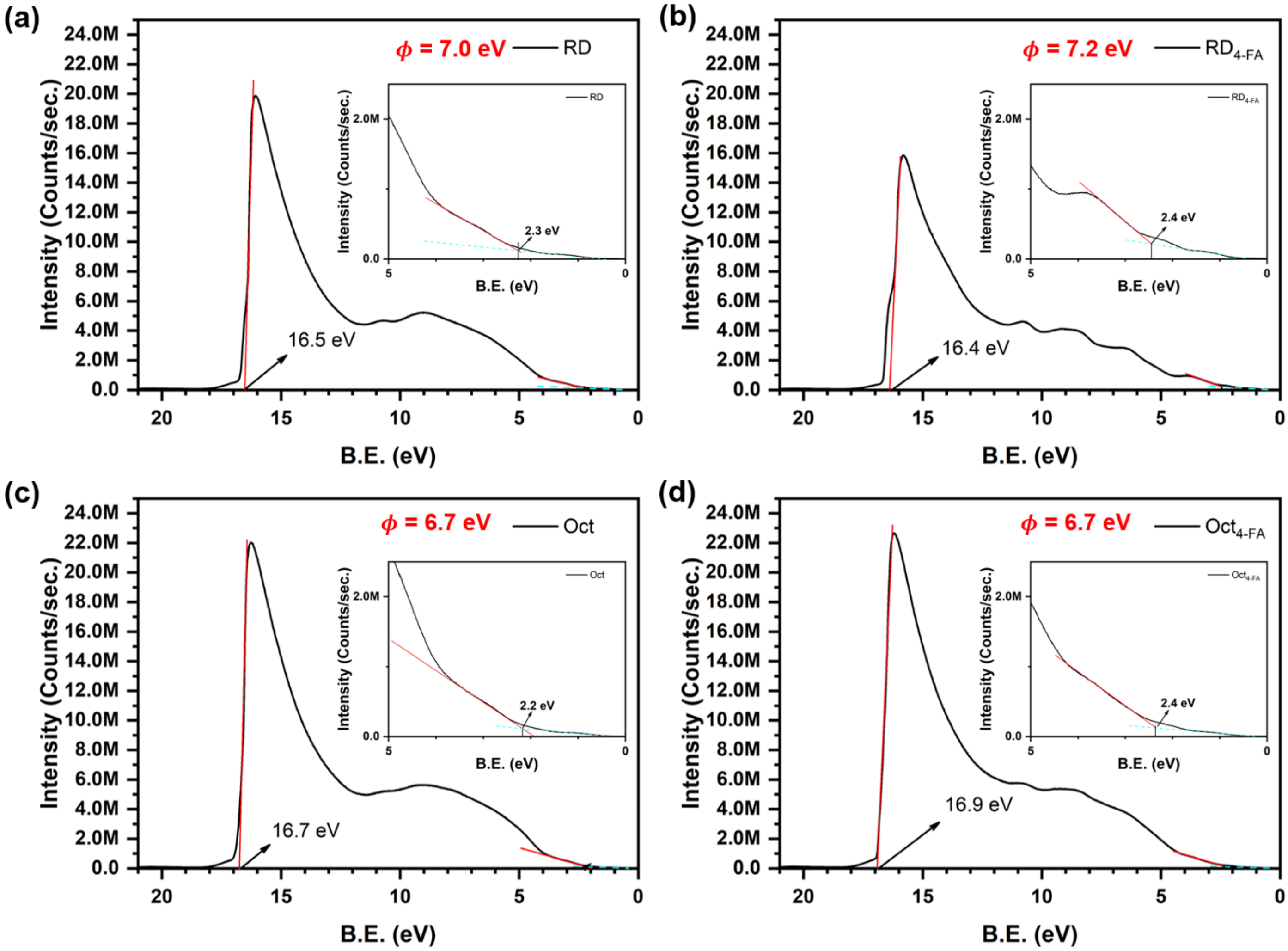

Figure S12. UPS spectra of the (a) rhombic dodecahedra, (b) 4-FA-modified rhombic dodecahedra, (c) octahedra, and (d) 4-FA-modified octahedra.
